# A gapless genome of early-diverging Asteraceae species, *Gerbera*, provides insights for ray floret differentiation in the capitula

**DOI:** 10.1093/hr/uhaf357

**Published:** 2025-12-09

**Authors:** Xiaohui Wen, Fan Li, Chunlian Jin, Qinli Shan, Chunmei Yang, Bohao Wang, Fuhui Sun, Qiang Gao, Huichun Liu, Xiaofan Zhou, Kaiyuan Zhu, Liangsheng Zhang, Shenchong Li

**Affiliations:** Zhejiang Institute of Landscape Plants and Flowers, Zhejiang Academy of Agricultural Sciences, Hangzhou 311251, China; Floriculture Research Institute, Yunnan Academy of Agricultural Sciences, National Engineering Research Center for Ornamental Horticulture, Key Laboratory for Flower Breeding of Yunnan Province, Kunming 650205, China; Yunnan Seed Laboratory, Kunming 650205, China; Floriculture Research Institute, Yunnan Academy of Agricultural Sciences, National Engineering Research Center for Ornamental Horticulture, Key Laboratory for Flower Breeding of Yunnan Province, Kunming 650205, China; Yunnan Seed Laboratory, Kunming 650205, China; Floriculture Research Institute, Yunnan Academy of Agricultural Sciences, National Engineering Research Center for Ornamental Horticulture, Key Laboratory for Flower Breeding of Yunnan Province, Kunming 650205, China; Floriculture Research Institute, Yunnan Academy of Agricultural Sciences, National Engineering Research Center for Ornamental Horticulture, Key Laboratory for Flower Breeding of Yunnan Province, Kunming 650205, China; Beijing Key Laboratory of Ornamental Plants Germplasm Innovation & Molecular Breeding, National Engineering Research Center for Floriculture, Beijing Laboratory of Urban and Rural Ecological Environment, Key Laboratory for Genetics and Breeding of Forest Trees and Ornamental Plants of Ministry of Education, School of Landscape Architecture, Beijing Forestry University, Beijing 100083, China; Zhejiang Key Laboratory of Horticultural Crop Quality Improvement, College of Agriculture and Biotechnology, Zhejiang University, Hangzhou 310058, China; Zhejiang Key Laboratory of Horticultural Crop Quality Improvement, College of Agriculture and Biotechnology, Zhejiang University, Hangzhou 310058, China; Zhejiang Institute of Landscape Plants and Flowers, Zhejiang Academy of Agricultural Sciences, Hangzhou 311251, China; Guangdong Laboratory for Lingnan Modern Agriculture, Guangdong Province Key Laboratory of Microbial Signals and Disease Control, Integrative Microbiology Research Center, South China Agricultural University, Guangzhou 510642, China; Zhejiang Institute of Landscape Plants and Flowers, Zhejiang Academy of Agricultural Sciences, Hangzhou 311251, China; Zhejiang Key Laboratory of Horticultural Crop Quality Improvement, College of Agriculture and Biotechnology, Zhejiang University, Hangzhou 310058, China; Floriculture Research Institute, Yunnan Academy of Agricultural Sciences, National Engineering Research Center for Ornamental Horticulture, Key Laboratory for Flower Breeding of Yunnan Province, Kunming 650205, China; Yunnan Seed Laboratory, Kunming 650205, China

Dear Editor,

*Gerbera hybrida* is not only one of the most commercially significant cut flowers in the worldwide, but also serves as an ideal model for studying Asteraceae biodiversity. *Gerbera hybrida* belongs to Mutisieae, an early-diverging lineage of Asteraceae that forms a sister group to Barnadesioideae [1]. It serves as both an essential outgroup for Asteroideae and an evolutionary transitional group bridging the basal Barnadesioideae and Asteroideae, providing crucial insights into Asteraceae evolution and diversification [1, 2]. Notably, the capitulum is regarded as a key evolutionary innovation in Asteraceae, consisting of disk florets, ray florets, or both. Compared with ray florets of early-diverging Asteraceae clades, ray florets of *Gerbera* display distinct zygomorphic symmetry and morphological diversification, showing convergent evolution with Asteroideae. Thus, uncovering the molecular regulatory networks underlying ray floret differentiation in *Gerbera* holds significant value for deciphering the adaptive evolution of ray florets in Asteraceae. However, the complex genetic background of *G. hybrida* remains unclear, which has hindered studies on Asteraceae evolution and genetic breeding of *G. hybrida*.

We performed whole-genome sequencing on *G. hybrida* ‘sh6,’ an ovule-derived haploid line. A total of 120 Gb (~50× coverage) of PacBio HiFi reads was generated, and assembled into a 2.40-Gb *Gerbera* genome with a contig N50 length of 74.18 Mb. We then anchored the scaffolds onto 25 pseudochromosomes, scaffold N50 up to 86.82 Mb (Fig. 1A, Supplementary Data Fig. S1, Supplementary Data Table S1). The assembled genome contains only 33 gaps, comprising 8 gap-free chromosomes, 11 chromosomes with 1 gap and 6 chromosomes with no more than 3 gaps (Supplementary Data Table S2). Only chromosome 4 contains 10 gaps. These results confirm that we have obtained a gapless genome of *Gerbera*. In addition, long terminal repeats (LTRs) were the most abundant repetitive sequence type, accounting for ~50.95% of the *Gerbera* genome, with 34.64% *copia* and 8.82% *Gypsy* (Supplementary Data Table S3). Benchmarking Universal Single-Copy Orthologs (BUSCO) analysis revealed 99% completeness of the annotated *Gerbera* genome, demonstrating the high quality of genome annotation (Supplementary Data Fig. S2).

Using the genomes of 24 representative angiosperm species to construct maximum likelihood phylogenetic trees, we found that *Gerbera* is positioned at the base of Asteraceae, forming a separate branch (Fig. 1B). The divergence time between *G. hybrida* and other Asteraceae plants was ~49.8 Mya (Fig. 1B). Among currently genome-sequenced Asteraceae species, *G. hybrida* occupies the most basal phylogenetic position. Gene family expansion and contraction analyses revealed 2581 gene family expansions and 1445 gene family contractions in *G. hybrida*, along with 1322 species-specific gene families (Supplementary Data Fig. S3). The synonymous substitutions per synonymous site (*K*_s_) for intraspecific and interspecific comparisons indicate that the *Gerbera* genome has undergone the ancient γ whole-genome triplication (WGT-γ) shared by core eudicots and the asterids II-specific whole-genome triplication (WGT-1) (Fig. 1B, Supplementary Data Fig. S4). Ancestral karyotype reconstruction revealed that the *Gerbera* genome underwent 32 fissions and 70 fusions to form the present 25 chromosomes (Supplementary Data Fig. S5). The maximum likelihood (ML) tree and genetic structure analysis divided the 49 *Gerbera* individuals into three clades (Fig. 1C, Supplementary Data Fig. S6 and S7). The genetic background of ‘sh6’ is present in >50% of the 49 individuals (Fig. 1C). Among them, ‘Chengsi,’ ‘Hongyun 16’ and ‘Xianggelila’ exhibit a highly similar genetic background to ‘sh6’ (Fig. 1C).

**Figure 1:**
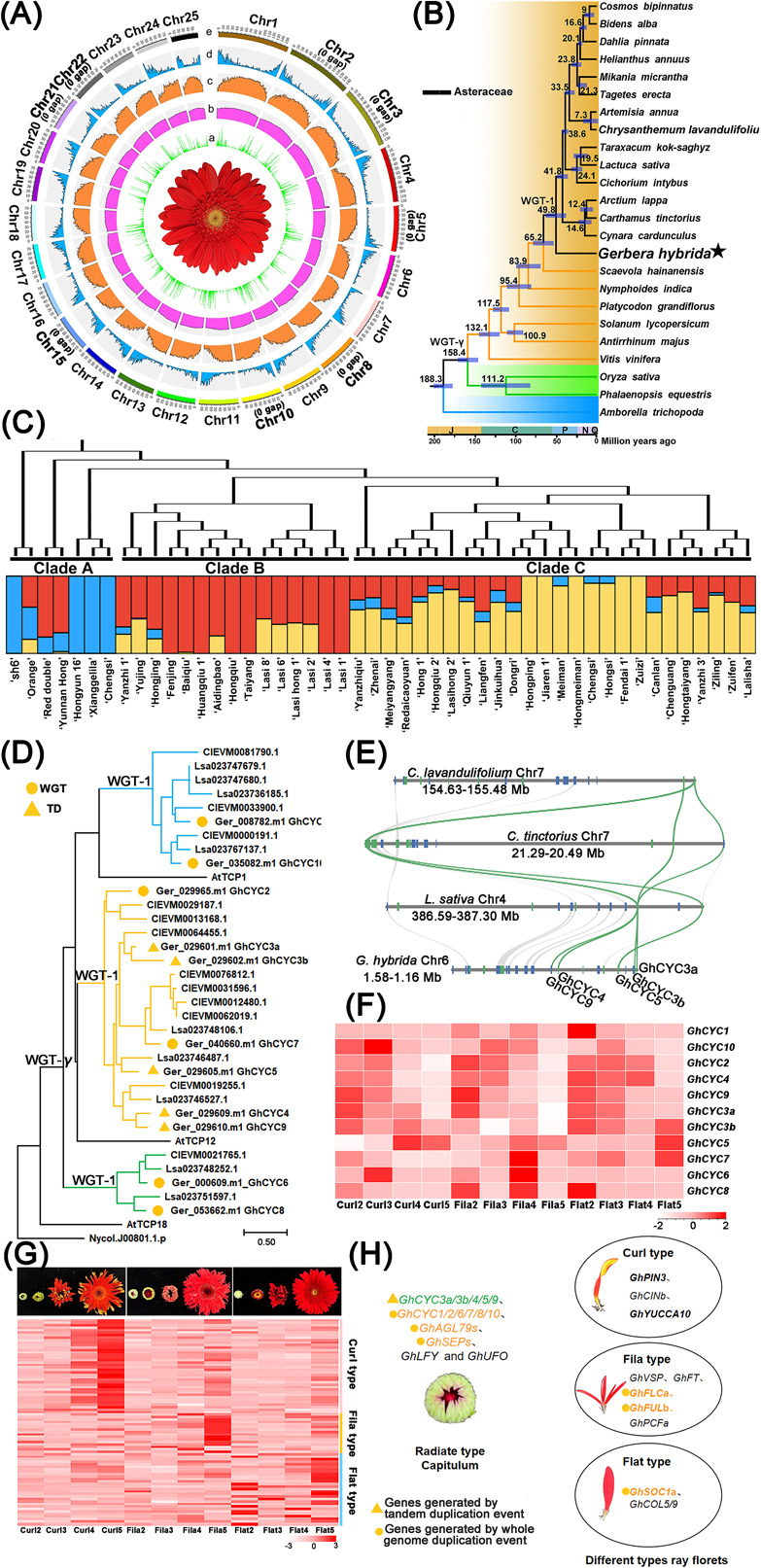
Multi-dimensional analysis of genome evolution and molecular mechanism underlying ray floret differentiation in *G. hybrida*. (A) Chromosomal-level assembly of *G. hybrida*. (a) Gene expression pattern in flat ray florets. (b) Repeat distribution in 1-Mb sliding windows. (c) Gene density in 1-Mb sliding windows. (d) GC content in 1-Mb sliding windows. (e) Chromosome size. (B) Phylogenetic tree using single-copy ortholog genes of 24 plant species dated with fossil calibrations. (C) Phylogenetic tree and genetic structure of 49 *Gerbera* individuals. (D) Gene duplication in TCP gene family of *G. hybrida*. (E) Collinear synteny of tandem-duplicated *CYC2-like* genes between *G. hybrida*, *L. sativa*, *C. tinctorius* and *C. lavandulifolium*. (F) Expression heap map of CYC genes generated by tandem duplications. Scale, log_2_^fpkm^. (G) Candidate genes involved in curled, filamentous and flat ray floret development. (H) Genes potentially involved in capitulum development and ray floret differentiation. Genes in bold type are key regulators in ray floret differentiation.

Functional studies of some TCP and MADS-box genes have proved their important roles in regulating capitulum development in *Gerbera* [3–5]. Phylogenetic analysis revealed the expansion of the TCP and MADS-box families in *G. hybrida*, which was mainly driven by the WGT-γ and WGT-1 (Supplementary Data Figs S8–S12). Notably, *GhCYC3a*, *GhCYC3b*, *GhCYC5*, *GhCYC4*, and *GhCYC9* were generated by tandem duplication (TD) events (Fig. 1D and E). A TD event also occurred in the CIN clade (Supplementary Data Fig. S13). These TD events in the TCP gene family were shared in Asteroideae and Mutisieae (Fig. 1D and E). Based on genomic and transcriptomic data, the present study reveals the expression divergence of duplicated genes in the TCP and Type II MADS-box gene families (Supplementary Data Figs S14 and S17). Tandem-duplicated genes (*GhCYC3a* and *GhCYC3b*, *GhCYC4*, and *GhCYC9*) exhibited relatively consistent expression patterns, suggesting potential functional redundancy of their roles in ray floret differentiation (Fig. 1F, Supplementary Data Fig. S18), while the expression of TD gene *GhCYC5* peaked at stage 2–3 of different types of ray florets, differing from the other four tandem duplicates (Fig. 1F, Supplementary Data Fig. S18). Furthermore, we found that *GhPCFa* (*Ger_047834.m1*) showed high expression at stage 2 and then decreased during filamentous ray floret development (Supplementary Data Figs S14 and S18). Collinear orthologs of *GhPCFa* were identified in *A. lappa*, *L. sativa*, *P. grandiflorus*, and *S. hainanensis*, suggesting its conserved functional role in the order Asterales (Supplementary Data Fig. S19). *GhCINa* (*Ger_002727.m1*) was highly expressed in the curled ray florets (Figs S14 and S18). The collinear orthologs of *GhCINa* originated from an ancient eudicot and were retained in Asteraceae (Supplementary Data Fig. S20). These two genes might also serve as crucial genes in ray floret differentiation, particularly in determining ray floret morphology. Duplicated genes in the MADs-box gene family also exhibited divergent expression patterns in different types of ray floret (Supplementary Data Figs S15 and S16). Most of the MADs-box genes showed similar expression patterns across different types ray floret (Supplementary Data Figs S15 and S16). Exceptions were *GhFLCa* (*Ger_000067.m1*) and *GhFULa* (*Ger_008738.m1*), which were expressed highly at stage 5 during the development of filamentous ray florets (Supplementary Data Figs S21 and S22). *GhSOC1a* (*Ger_047828.m1* subfamily) also showed a high expression level at stage 5 during the development of curled and flat ray florets, especially the flat ray florets (Supplementary Data Fig. S22).

To better understand the molecular mechanism underlying the development of different types of ray floret, we carried out weighted gene coexpression network analysis (WGCNA) using transcriptomic data on flat, filamentous and curled type ray florets (Fig. 1G, Supplementary Data Fig. S23). A total of 25 401 expressed genes were clustered into 13 color modules (Supplementary Data Fig. S23). Genes expressed in the yellow color module were correlated with curled type ray florets, and we found *Gh*NAC50/83/92, *GhCOb*, and *GhYUCCA10* were highly expressed in curled type ray florets compared with low expression level of those genes in filamentous and flat ray florets (Supplementary Data Fig. S24). Filamentous ray florets were regulated by genes clustered into the pink color module, including *GhVSP*, *GhFT* and so on (Supplementary Data Fig. S25). Genes expressed in the red color module were correlated with flat ray florets, and were represented by *GhSOC1a* and *GhCOL5/9* (Supplementary Data Fig. S26). Finally, we obtained a total of 84 genes that might be involved in the formation of different types of ray floret (Fig. 1G, Supplementary Data Table S4). Based on the results of the coexpression network analysis, 16 genes were found to exhibit coexpression regulatory relationships (Supplementary Data Fig. S27). Among these, auxin-related genes *GhYUCCA10* (*Ger_054474.m1*) and *GhPIN3* (*Ger_053507.m1*) might participate in regulating the development of curled ray florets, while MADs-box genes *GhFLCa* (*Ger_000067.m1*) and *GhSOC1a* (*Ger_047828.m1*) may be involved in the development of filamentous and flat ray florets, respectively (Fig. 1H, Supplementary Data Fig. S27).

In summary, we report a 2.40-Gb chromosomal-level genome of *G. hybrida* ‘sh6’, with contig N50 74.18 Mb. Multiomics analyses revealed that gene duplication and expression divergence in the TCP and MADS-box gene families play important roles in ray floret differentiation. Additionally, *GhYUCCA10* and *GhPIN3* may also participate in curled ray floret development. Our study not only provides insights into Asteraceae evolution, but also primarily clarifies the molecular mechanism underlying ray floret differentiation, with benefits for the molecular breeding of *Gerbera*.

## Supplementary Material

Web_Material_uhaf357

## Data Availability

All raw data are available in the National Genomics Data Center (https://ngdc.cncb.ac.cn/) under project number PRJCA042663. The genome assembly sequences and gene annotations are available at https://doi.org/10.6084/m9.figshare.29482379.v1. The supplementary data and methods generated in this study are available from the Dryad Digital Repository: http://datadryad.org/share/LINK_NOT_FOR_PUBLICATION/bZj6KHEhjd38SL_PwWlWDiXVFQ17za6wBStaI254aaQ.
