# Supplementary material for: A gapless genome of early-diverging Asteraceae species, Gerbera, provides insights for ray floret differentiation in the capitula
Source: Hortic Res. 2025 Dec 9;13(3):uhaf357. doi: 10.1093/hr/uhaf357 (PMC13101651; doi:10.1093/hr/uhaf357)
Supplement: Web_Material_uhaf357 [file Web_Material_uhaf357.zip › Web_Material_uhaf357.pdf]

A gapless genome of early-diverging Asteraceae species Gerbera provides insights for ray florets differentiation in the capitula

Xiaohui Wen[1#], Fan Li[2,3,#], Chunlian Jin[2,3,#], Qinli Shan[2], Chunmei Yang[2], Bohao Wang[5], Fuhui Sun[4], Qiang Gao[4], Huichun Liu[1], Xiaofan Zhou[6], Kaiyuan Zhu[1,*],Liangsheng Zhang[3,4,*], Shenchong Li[2,3,*]

[1]Zhejiang Institute of Landscape Plants and Flowers, Zhejiang Academy of Agricultural Sciences, Hangzhou 311251, China.

[2]Floriculture Research Institute, Yunnan Academy of Agricultural Sciences, National Engineering Research Center for Ornamental Horticulture, Key Laboratory for Flower Breeding of Yunnan Province, Kunming 650205, China

[3]Yunnan Seed Laboratory, Kunming 650205, China

[4]Zhejiang Key Laboratory of Horticultural Crop Quality Improvement, College of Agriculture and Biotechnology, Zhejiang University, Hangzhou 310058, China.

[5]Beijing Key Laboratory of Ornamental Plants Germplasm Innovation & Molecular Breeding, National Engineering Research Center for Floriculture, Beijing Laboratory of Urban and Rural Ecological Environment, Key Laboratory for Genetics and Breeding of Forest Trees and Ornamental Plants of Ministry of Education, School of Landscape Architecture, Beijing Forestry University, Beijing 100083, China

[6]Guangdong Laboratory for Lingnan Modern Agriculture, Guangdong Province Key Laboratory of Microbial Signals and Disease Control, Integrative Microbiology Research Center, South China Agricultural University, Guangzhou 510642, China.

For Review Only

Supplementary Methods

Matrials and methods

Genomic DNA and transcriptomic RNA extraction, and sequencing

Genomic DNA was extracted from fresh and young leaves of *Gerbera hybrida* ‘sh6’ (Li et al., 2020) which planted in YunNan Province using QIAamp DNA Mini Kit/DNeasy Plant Mini Kit (QIAGEN). The PacBio DNA library was established and sequenced on the Sequel platform using the CCS mode followed by the PacBio’s standard protocol for third-generation sequencing. Long sequencing reads were

initially corrected to obtain sub-reads. And then the sub-reads were merged to generated the HiFi reads.

The plant organ tissues of *G. hybrida* was collected by manual dissection and frozen immediately in liquid nitrogen and stored at -80 °C before processing. Total RNA was extracted using Trizol regent (Invitrogen) method and were sequenced to generate 100-bp-long paired-end reads on an Illumina HiSeq2000 (Novogene).

Hi-C library construction and sequencing

After formaldehyde fixation and lysis for the young leaves of *G. hybrida*, the cross-linked DNA was digested with MboI, yielding DNA fragments of 500 - 700 bp in size. The Hi-C library was sequenced by using Illumina HiSeq X Ten system, and subsequently employed for anchoring scaffolds onto chromosomes (Burton et al., 2013).

Genome assembly and assessment

HiFi PacBio sequencing reads were assembled into contigs using hifiasm (v0.9) (https://github.com/chhylp123/hifiasm) (Cheng et al., 2021). Chromosome-scale scaffolds were generated using 3D-DNA (v 180922) and manually curated using JuiceBox (v 2.0) (Dudchenko et al., 2017; Durand et al., 2016). The completeness of the genome assembly was assessed using BUSCO (v5.4.0) with the embryophyta_odb10 (Simao et al., 2015).

Repeat annotation

To annotated repeat sequences of *G. hybrida* genome, a de novo repeat library was constructed using RepeatModeler (http://www.repeatmasker.org/RepeatModeler/). Then the library was integrated with Repbase (http://www.girinst.org/repbase), a database of known repetitive elements, to generate a comprehensive repetitive database (Bao et al., 2015). RepeatMasker (v4.1.5) was then used to identify repetitive sequences of *G. hybrida* with default parameters (Tarailo-Graovac and Chen, 2009).

Fusion and Breakage Events of Gerbera Genome.

The ancestral chromosomes AEK (Ancestral Eudicot Karyotype) identified in previous study (Murat et al., 2017) were aligned with the genes of each species to

For Review Only

determine the ancestral chromosomal origin of genes in Gerbera genome. These genes were then visualized according to the color scheme of their corresponding ancestral chromosomes. Genes without ancestral chromosome assignments were excluded. A genomic region was defined as a block if it contained at least 15 consecutive genes derived from the same ancestral chromosome. The number of fusion events for each chromosome was calculated as the number of blocks minus 1. The number of breakage events was determined based on the number of blocks derived from each ancestral chromosme and the WGD events. The number of breakage events was calculated as the observed number of blocks in the current genome minus the theoretically expected number after WGD events.

Genome evolution analysis

The WGD events in the genome of *G. hybrida* through synonymous substitution rate (*Ks*) distribution analysis. Protein sequences of *G. hybrida*, *H. annuus* and *V. vitis* were analyzed using DIAMOND v.0.9.26 for alignment with e-value cutoff of 1e-5 (Buckfink et al., 2015). Subsequently, the *Ks* values for each orthologouks gene pair were calculated using the Condeml program of the PAML package (Yang et al., 1997). The syntenic gene pairs were identified and visualized by using JCVI v.0.9.14 (https://pypi.org/project/jcvi/) (Tang et al., 2015). And the expansion and contraction of orthologous gene families in 24 species were analyzed using the software CAFE´ v.4.2.1 (https://github.com/hahnlab/CAFE) (Han et al., 2013).

Population Structure Analysis

Transcriptome sequencing data from 49 *G. hybrida* cultivars and the reference genome of *G. hybrida* ‘sh6’ were used for population structure analysis (Figure S6). Clean transcriptome sequencing reads from each of the 49 *Gerbera* cultivars were aligned to *G. hybrida* ‘sh6’ reference genome using HISAT2 (v2.2.1). Subsequently, the aligned BAM files were processed using GATK (Genome Analysis Toolkit) to ensure data quality and perform variant calling (Danecek et al., 2011). Variant quality control (QC) was performed using GATK VariantFiltration. The parameters were set as follows: --filter-expression "QD < 2.0 || FS > 60.0 || MQ < 40.0 || SOR > 3.0 || MQRankSum < -12.5 || ReadPosRankSum < -8.0", and --restrict-alleles-to

For Review Only

BIALLELIC was specified to retain only biallelic variants. VCFtools was used for further filtering with the parameters --max-missing 0.9 and --maf 0.05. High quality SNPs retained after QC were extracted from the final filtered VCF file and converted to PLINK binary format using PLINK (v1.90) (Purcell et al., 2007). The population structure was inferred using Admixture (v1.3.0). To determine the optimal K value, admixture analyses were run for K = 2 to K = 10 with 10 independent replicates per K (Tang et al., 2005). Each replicate was executed with the --cv flag to calculate cross-validation (CV) error, the optimal K was identified as the lowest CV error. Ancestry proportions from the optimal K were visualized using R (v4.3.1) with the ggplot2 package to generate an admixture plot.

Phylogenetic and gene family analysis

The protein sequences of MADS-box and TCP gene family were identity by HMMER 3.0 with default parameters using conserved domain of MADS-box (pfam00319 and pfam01486) and TCP (PF) (Camacho et al., 2009). Multiple sequence alignment was done by MAFFT v7.453 (Katoh and Standley, 2013), and phylogenetic gene trees were constructed using FastTree v2.1 (Price et al., 2009).

Transcriptome analysis

The clean reads were mapped to the *G. hybrida* genome using Hisat2 v 2.0.4 with default settings for parameters, after removing the adaptors and low-quality reads were removed (Kim et al., 2015). The bam files of uniquely mapped reads were used as inputs and FPKM (fragments per kilobase of transcript per million fragments) values were calculated to measure the expression levels of genes using Stringtie v 2.1.4 software (Kovaka et al., 2019). The WGCNA was performed using R packages with the 8 thresholding power to establish adjacency matrix of expressed genes (FPKM $\geq$ 1) (Langfelder and Horvath, 2008). The expressed genes were then hierarchically clustered and divided in different color modules using Dynamic Hybrida Tree Cut (minModuleSize = 30) (Langfelder and Horvath, 2008).

Reference

Bao, W., Kojima, K. K., and Kohany, O. Repbase Update, a database of repetitive elements in eukaryotic genomes. Mobile DNA. 2015; 6, 11.

For Review Only

Buchfink, B., Xie, C., and Huson, D.H. Fast and sensitive protein alignment using DIAMOND. Nature Methods. 2015; 12: 59-60.

Burton, J. N., Adey, A., Patwardhan, R. P. et al. Chromosome-scale scaffolding of de novo genome assemblies based on chromatin interactions. Nature Biotechnology. 2013; 31: 1119-1125.

Camacho, C., Coulouris, G., Avagyan, V. et al. BLAST+: architecture and applications. BMC Bioinformatics. 2009; 10: 421.

Cheng, H., Concepcion, G. T., Feng, X., Zhang, H. et al Haplotype-resolved de novo assembly using phased assembly graphs with hifiasm. Nature Methods. 2021; 18: 170-175.

Danecek, P., Auton, A., Abecasis, G. et al. The variant call format and VCFtools. Bioinformatics. 2011; 27: 2156-2158.

Dudchenko, O., Batra, S., Omer, A. D. et al. De novo assembly of the Aedes aegypti genome using Hi-C yields chromosome-length scaffolds. Science. 2017; 356: 92-95.

Durand, N. C., Robinson, J. T., Shamim, M. S. et al. Juicebox provides a visualization system for Hi-C contact maps with unlimited zoom. Cell Systems. 2016; 3: 99-101.

Katoh, K., and Standley, D. M. MAFFT multiple sequence alignment software version 7: improvements in performance and usability. Molecular Biology and Evolution. 2013; 30: 772-780.

Kim, D., Landmead, B., and Salzberg, S. L. HISAT: a fast spliced aligner with low memory requirements. Nature Methods. 2015; 12: 357-60.

Kovaka, S., Zimin, A.V., Pertea, G.M. et al. Transcriptome assembly from long-read RNA-seq alignments with StringTie2. Genome Biology. 2019; 20: 278.

Langfelder, P., and Horvath, S. WGCNA: an R package for weighted correlation network analysis. BMC Bioinformatics. 2008; 9: 559.

Li, F., Cheng, Y., Zhao, X. K. et al. Haploid induction via unpollinated ovule culture in *Gerbera hybrida*. Scientific reports. 2020; 10: 1702.

Murat, F. A., Armero, A., Pont, C. et al. Reconstructing the genome of the most recent common ancestor of flowering plants. Nature Genetics. 2017; 49: 490-496.

Price, M. N., Dehal, P. S., and Arkin, A. P. FastTree: computing large minimum evolution trees with profiles instead of a distance matrix. Molecular Biology and Evolution. 2009; 26: 1641-1650.

Purcell, S., Neale, B., Todd-Brown, K. et al. PLINK: a tool set for whole-genome association and population-based linkage analyses. The American Journal of Human Genetics. 2007; 81(3):

For Review Only

559-575.

Simão, F. A., Waterhouse, R. M., Ioannidis, P., et al. BUSCO: assessing genome assembly and annotation completeness with single-copy orthologs. Bioinformatics. 2015; 31: 3210-3212.

Tang, H. B., Krishnakumar, V., Zeng, X. F. et al. JCVI: A versatile toolkit for comparative genomics analysis. iMeta. 2024; 3: e211.

Tang, H., Peng, J., Wang, P. et al. Estimation of individual admixture: analytical and study design considerations. Genetic Epidemiology. 2005; 28: 289-301.

Tarailo-Graovac, M., and Chen, N. Using RepeatMasker to identify repetitive elements in genomic sequences. Curr Protoc Bioinformatics Chapter 4, 2009; Unit 4.10.

Yang, Z. PAML: a program package for phylogenetic analysis by maximum likelihood. Bioinformatics. 1997; 13: 555-556.

For Review Only

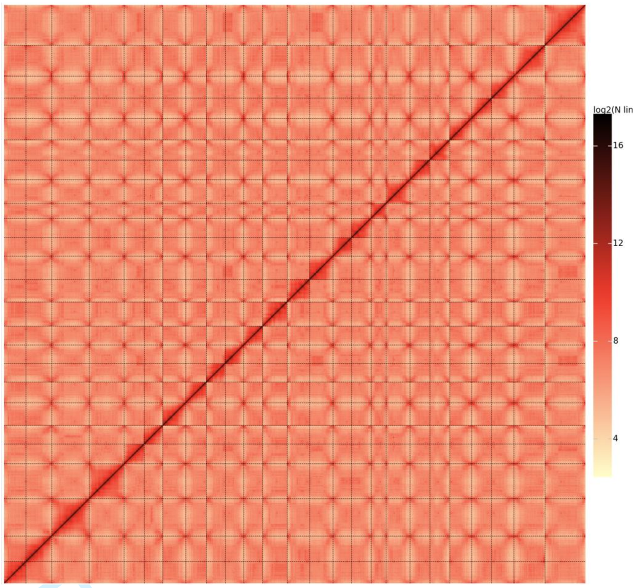

Figure S1 Hi-C heatmap for the chromosome-scale assembly of the *G. hybrida* genome

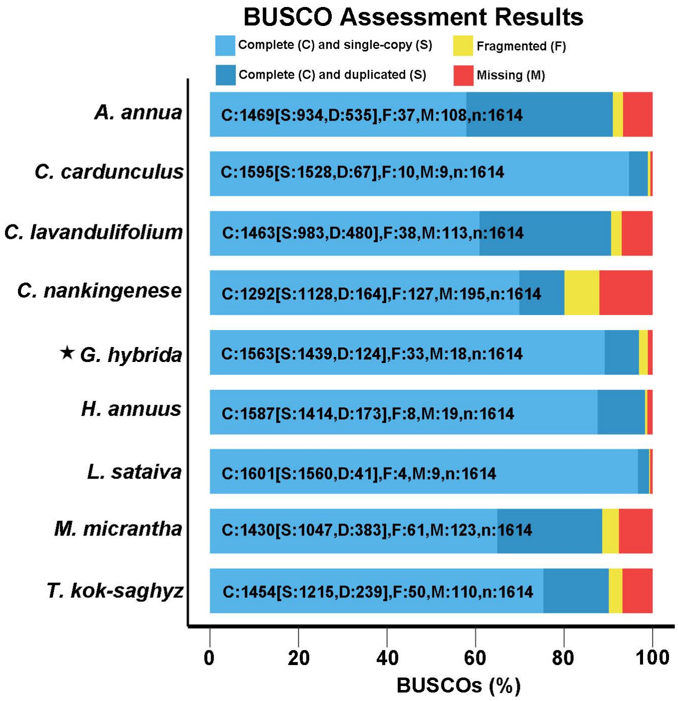

Figure S2 BUSCO assessment of protein-coding gene annotation for *G. hybrida* 'sh6' and other eight Asteraceae. *A. annuua*: *Artemisia annua*; *C. cardunculus*: *Cynara cardunculus*; *C. lavandulifolium*: *Chrysanthemum lavandulifolium*; *C. nankingenese*: *Chrysanthemum nankingenese*; *G. hybrida*: *Gerbera hybrida* 'sh6'; *H. annuus*: *Hellianthus annuus*; *L. sativa*: *Lactuca sativa*; *M. micrantha*: *Mikania micrantha*; *T. kok-saghyz*: *Taraxacum kok-saghyz*.

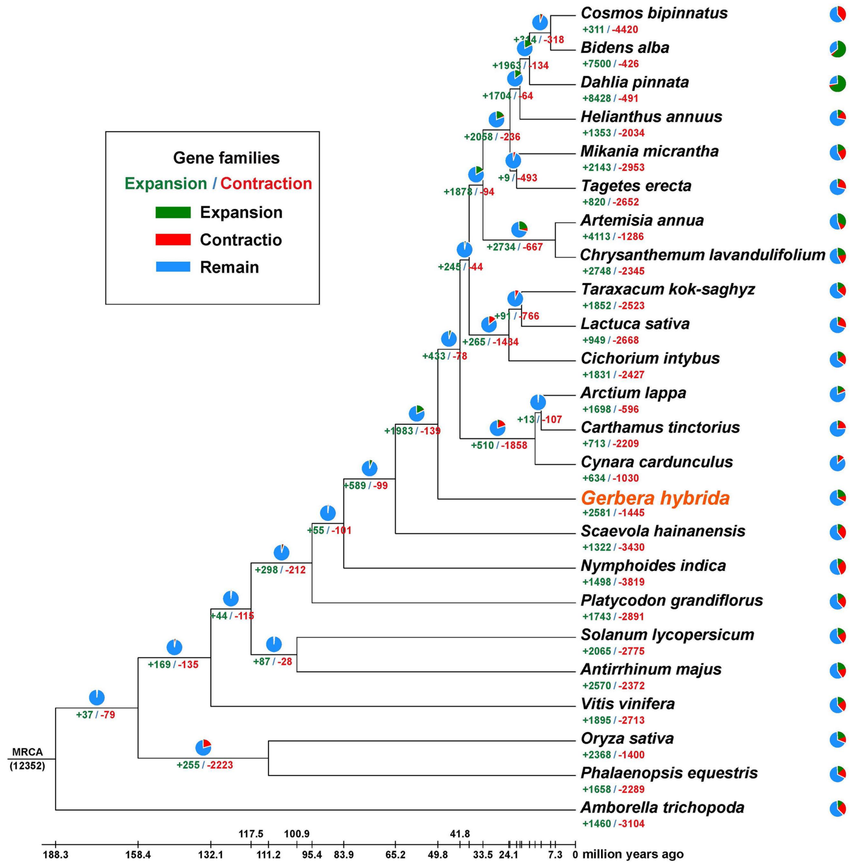

Figure S3 The gene families expansion and contraction among 24 representative species, including *Gerbera hybria*.

Review Only

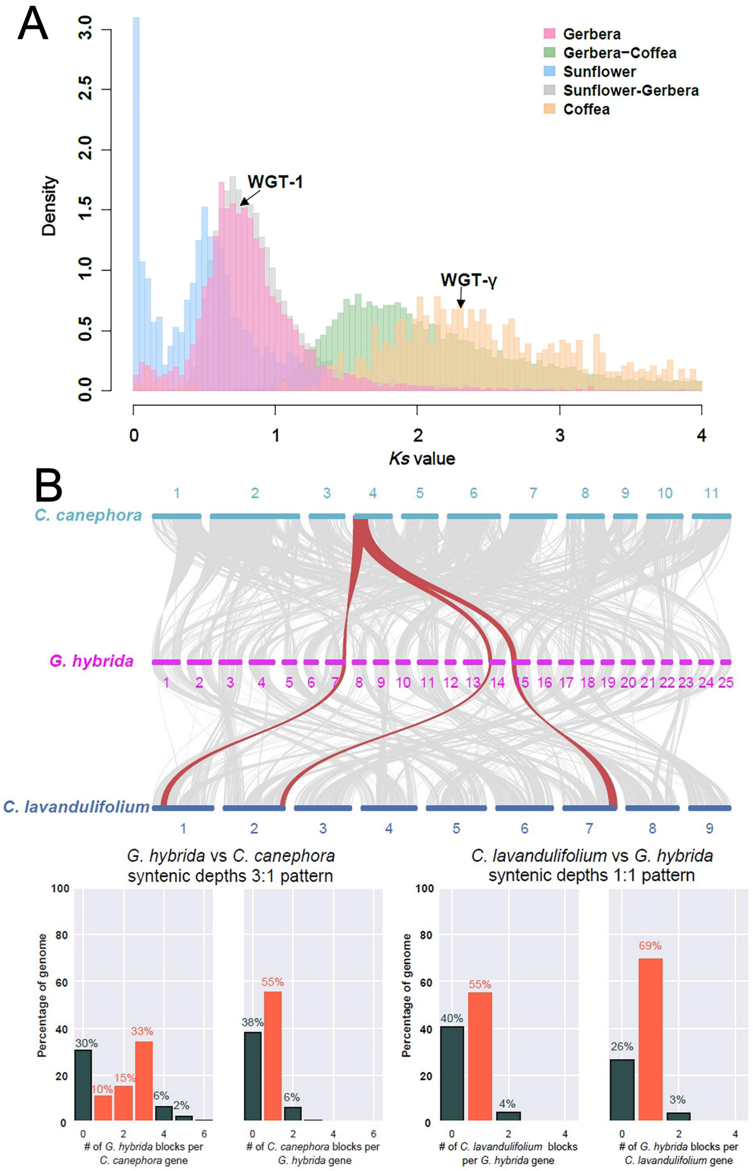

Figure S4 Evolution of Gerbera genome. A, *Ks* distribution for synteny gene pairs in Gerbera (*G. hybrida*), Sunflower (*H. annus*) and Coffea (*Coffea canephora*). B, Synteny analysis between genomic regions from *G. hybrida*, *C. canephora* and *C. lavandulifolium*.

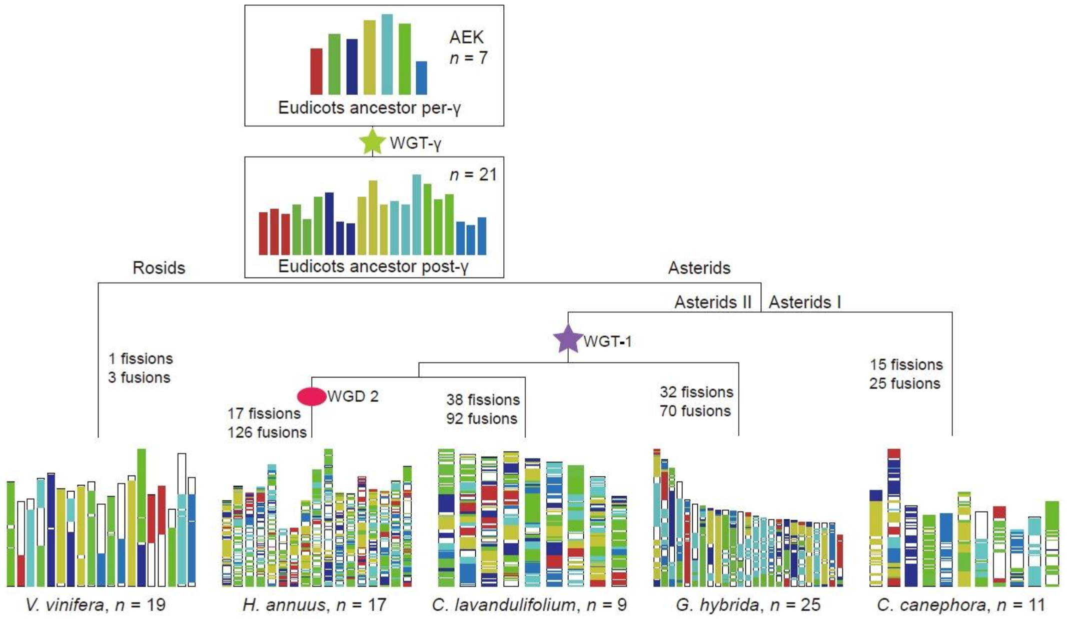

Figure S5 Ancestral Karyotype Reconstruction from the AEKs of 21 (post-WGT-γ) and 7 (pre-WGT-γ) protochromosomes to reveal the *G. hybrida* genome evolution. The modern genomes (bottom) are presented with different colors to reflect the origin from the seven ancestral chromosomes from the n=7 AEK (top).

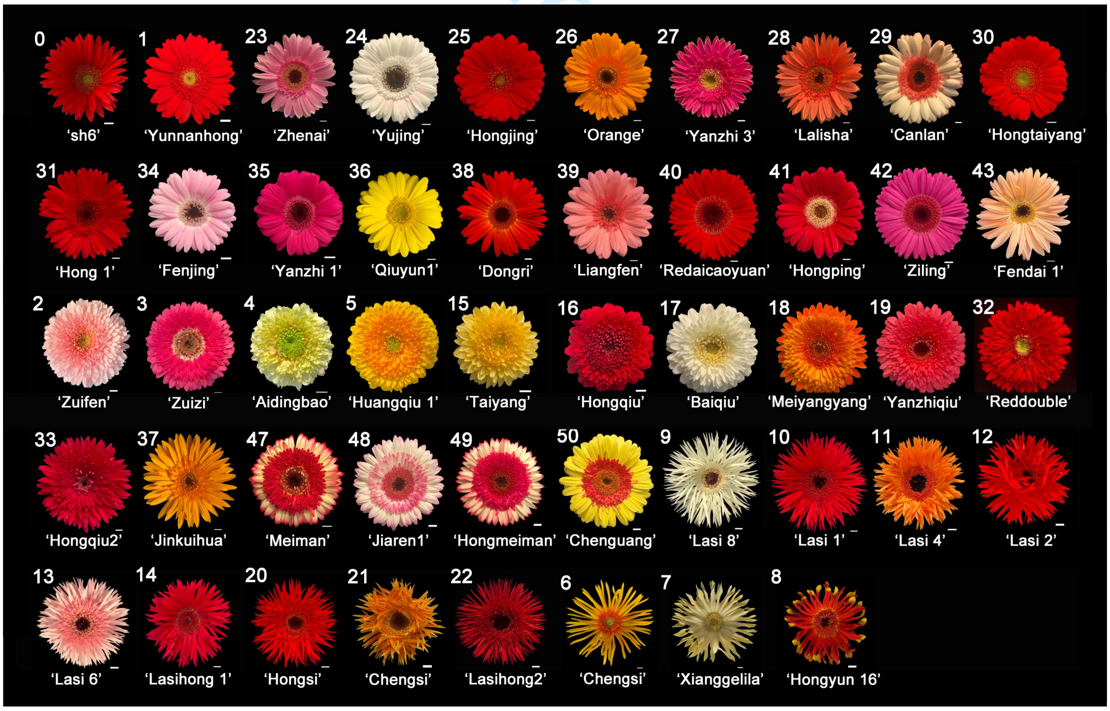

Figure S6 The 49 Gerbera individuals used for population genetic structure analysis.

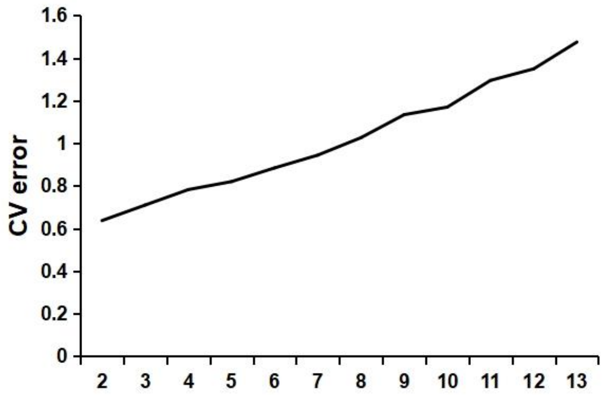

Figure S7 The CV error curve with different K-value for population genetic structure analysis of 48 Gerbera individuals

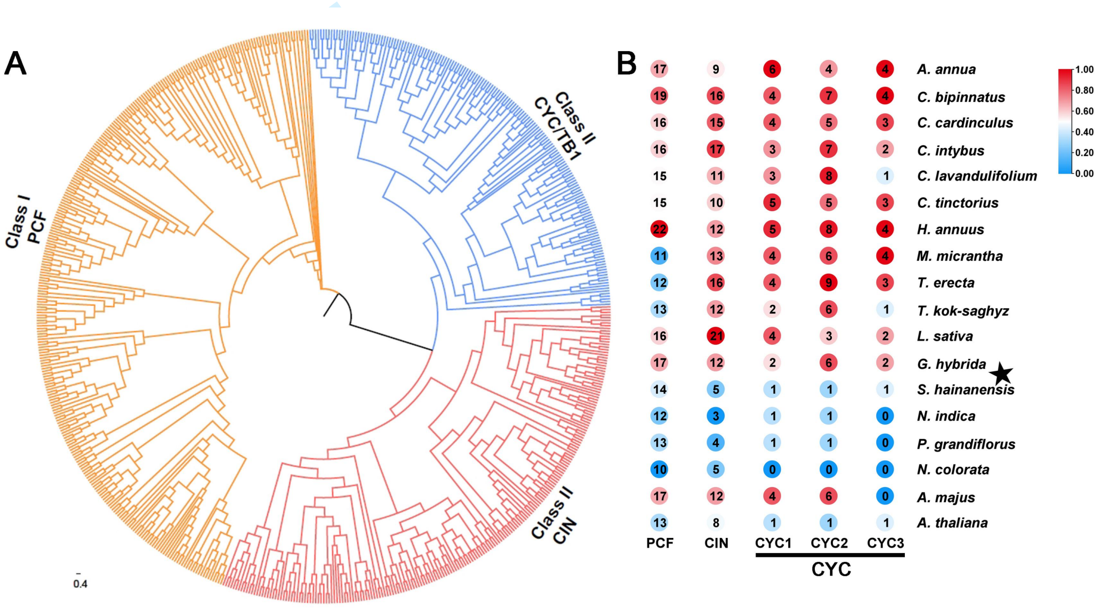

Figure S8 The classification and members statistics of TCP gene family. A, The phylogentic gene tree of TCP gene family in Asteraceae. B. the member statistics of TCP gene family in Asteraceae and other representative angiosperms.

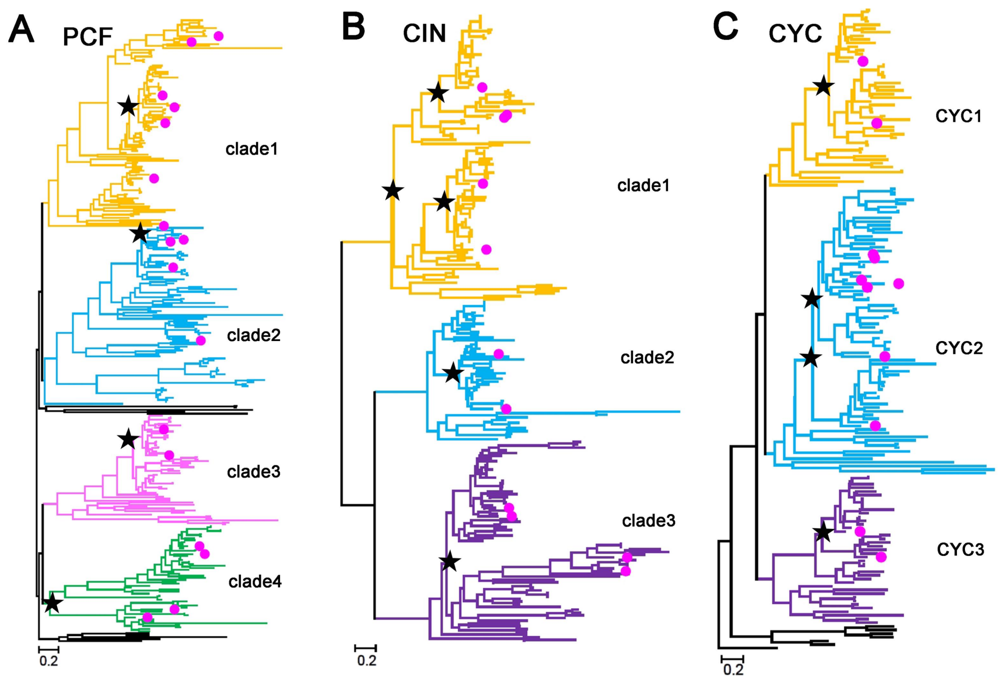

Figure S9 The phylogenetic gene trees of PCF, CIN and CYC subfamilies with marked genes in *G. hybrida* genome. Note: The magenta circles represent the genes in *G. hybrida* genome, the stars represent the occurrence of gene duplication.

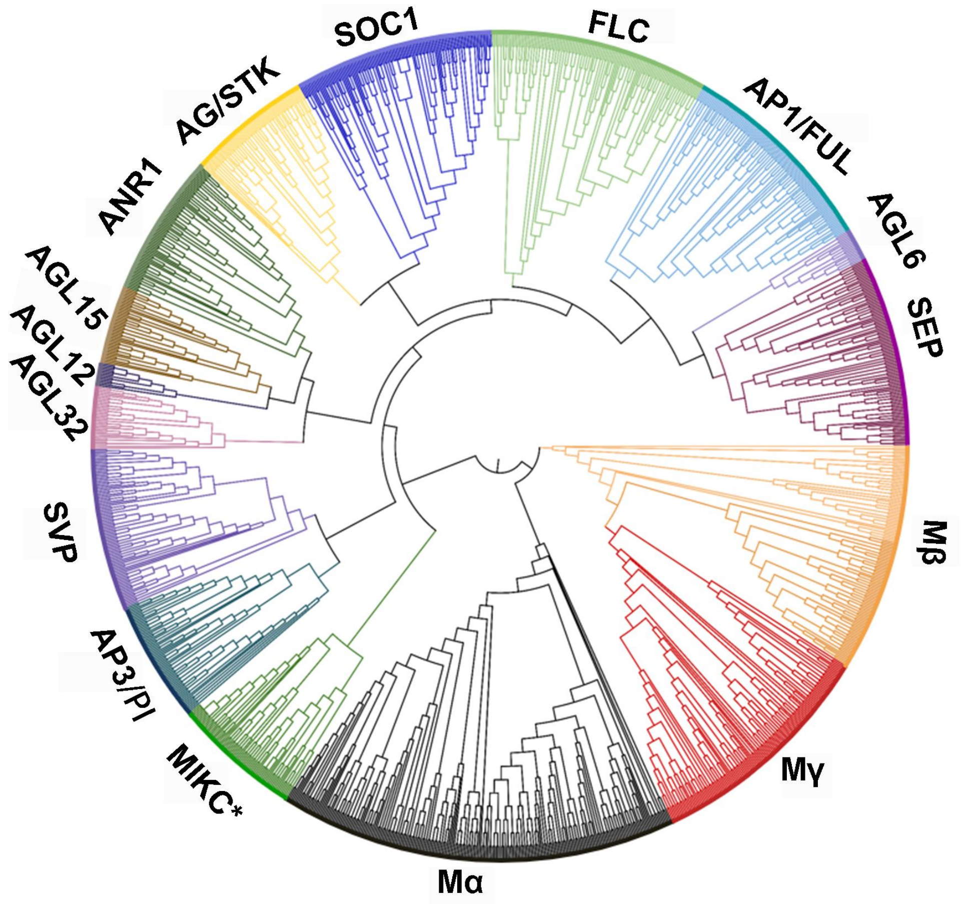

Figure S10 The phylogentic gene tree of MADS-box gene family in Asteraceae

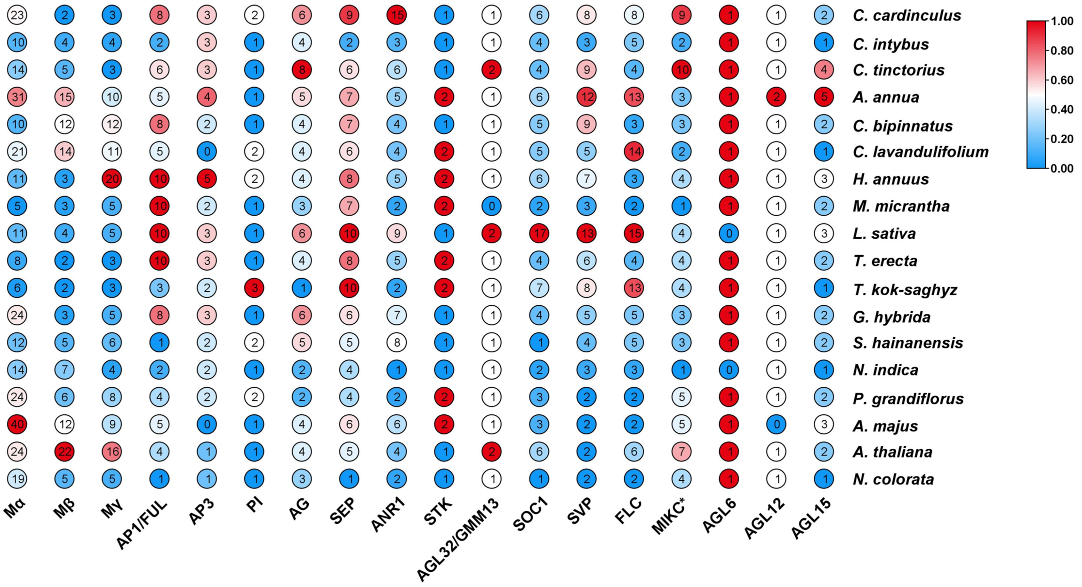

Figure S11 The members statistics of MADS-box gene family in Asteraceae and other representative angiosperms.

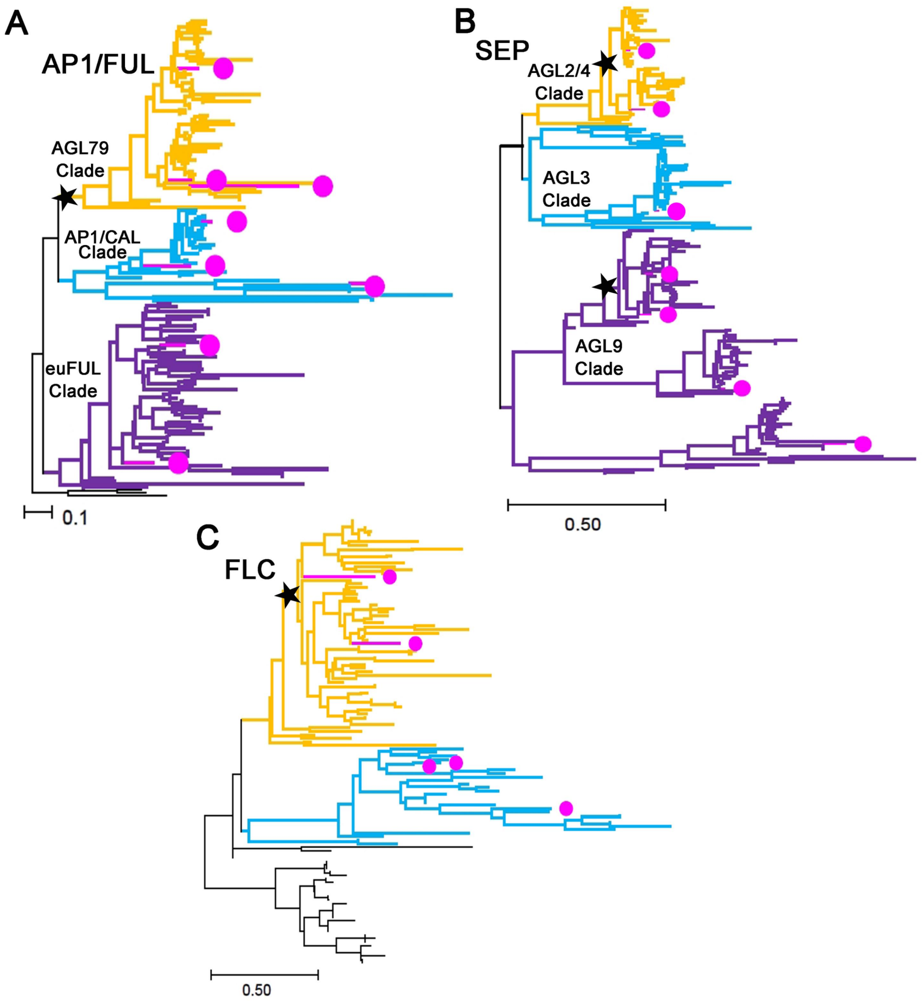

Figure S12 The phylogenetic gene trees of AP1/FUL, SEP and FLC subfamilies with marked genes in *G. hybrida* genome. Note: The magenta circles represent the genes in *G. hybrida* genome, the stars represent the occurrence of gene duplication.

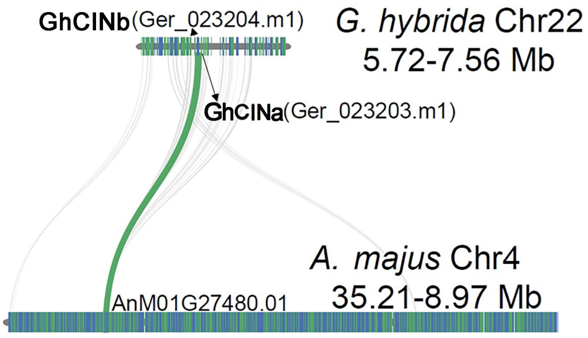

Figure S13 The collinear synteny of tandem duplicated CIN-like genes between *G. hybrida* and *A. majus*.

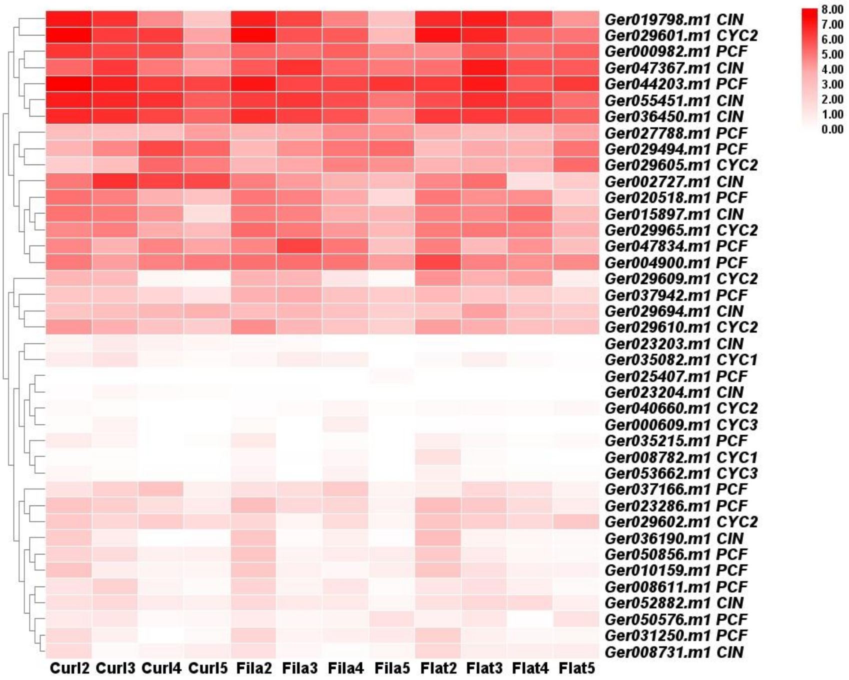

Figure S14 The heatmap of all genes belonged to TCP gene family. Scale: Log_2^{FPKM}.

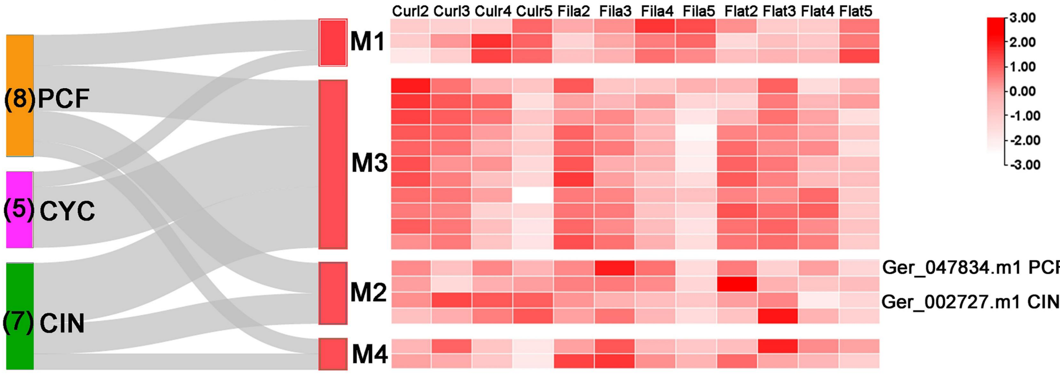

Figure S15 The expression divergence of genes in TCP gene family. The numbers in parentheses represent the number of genes expressed in the clade.

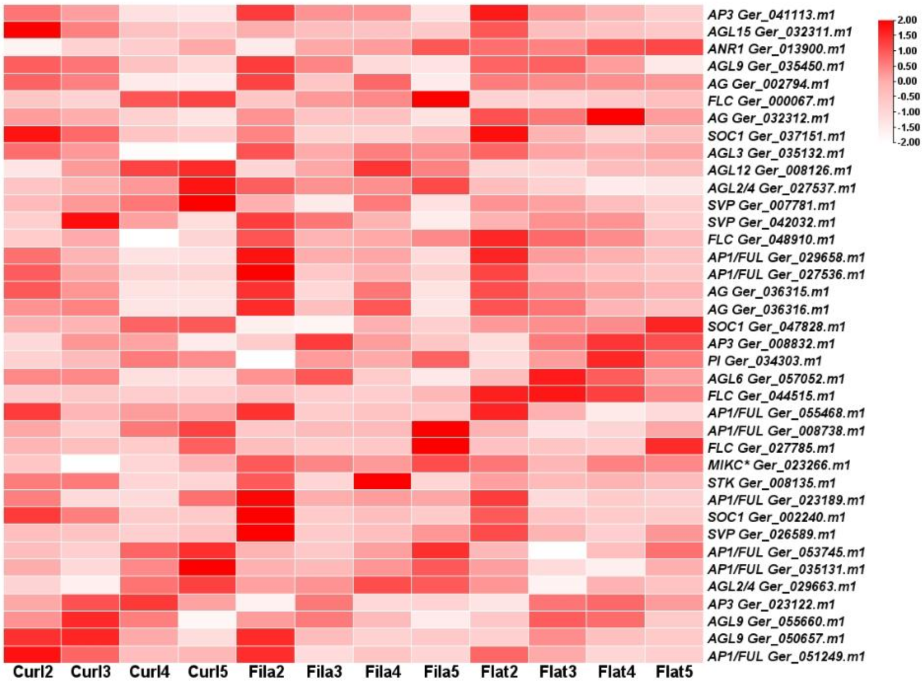

Figure S16 The heatmap of all genes belonged to MADS-box gene family. Scale: Log_2^{FPKM}.

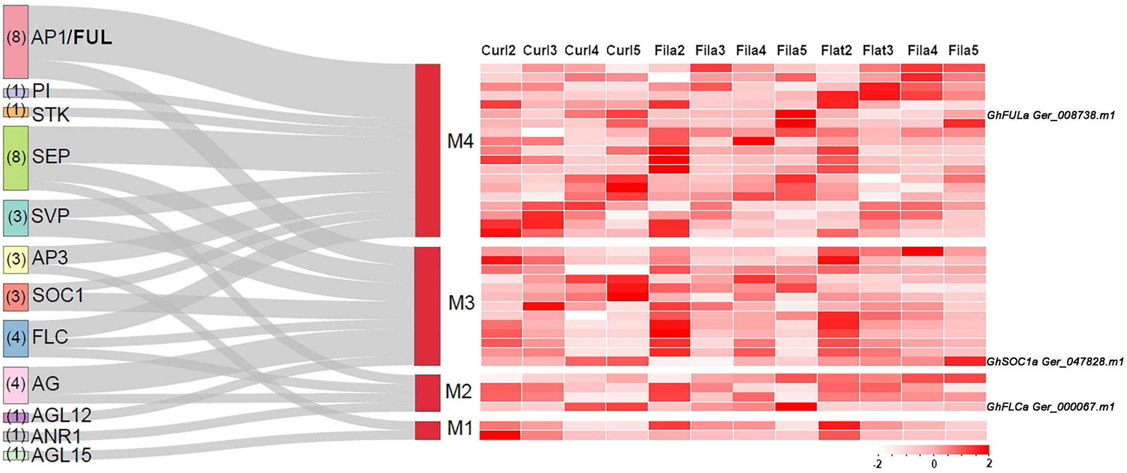

Figure S17 The expression divergence of genes in MADS-box gene family. The numbers in

parentheses represent the number of genes expressed in the clade.

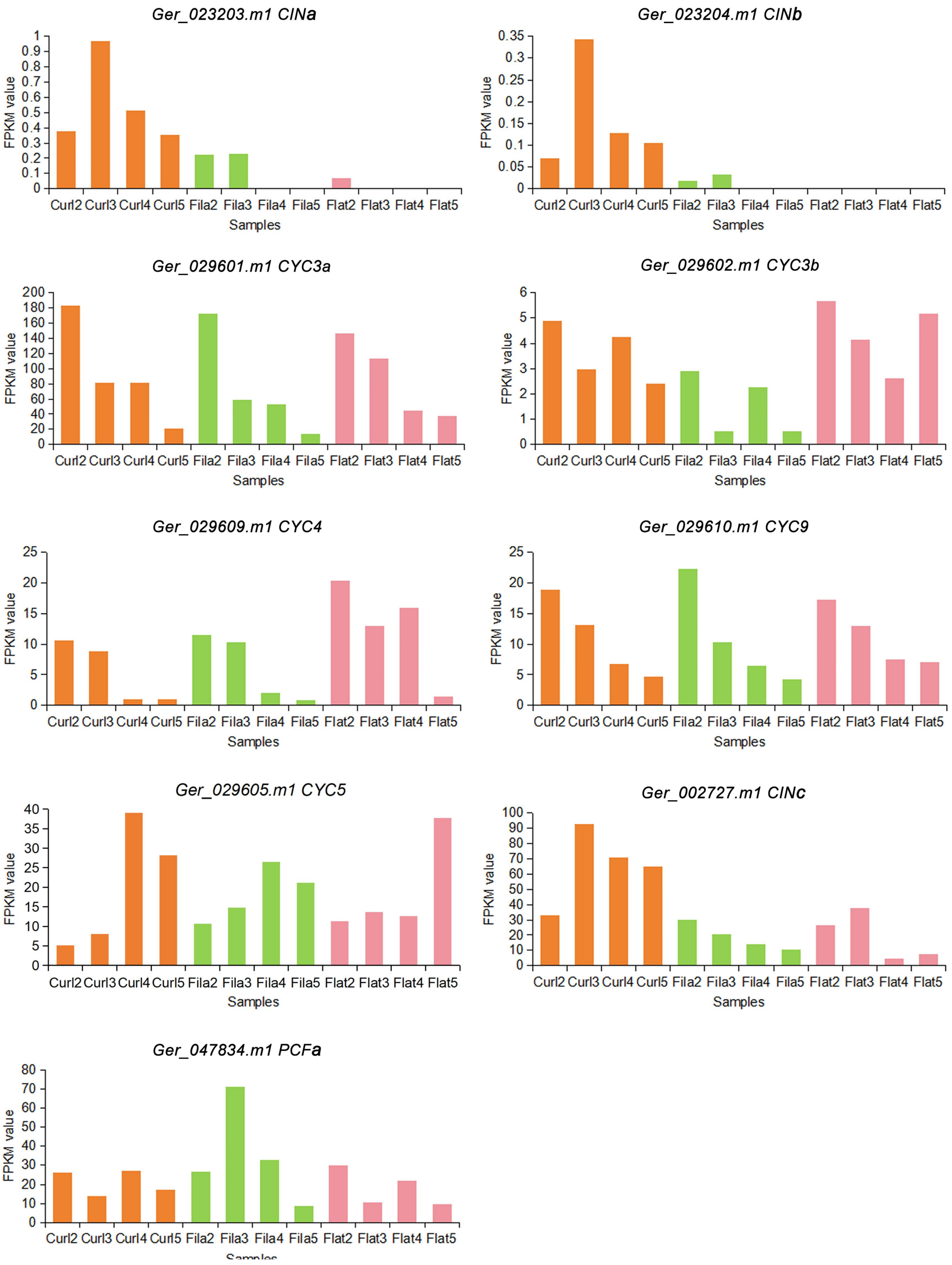

Figure S18 The expression patterns of tandem duplicated genes and key candidate genes in TCP gene family.

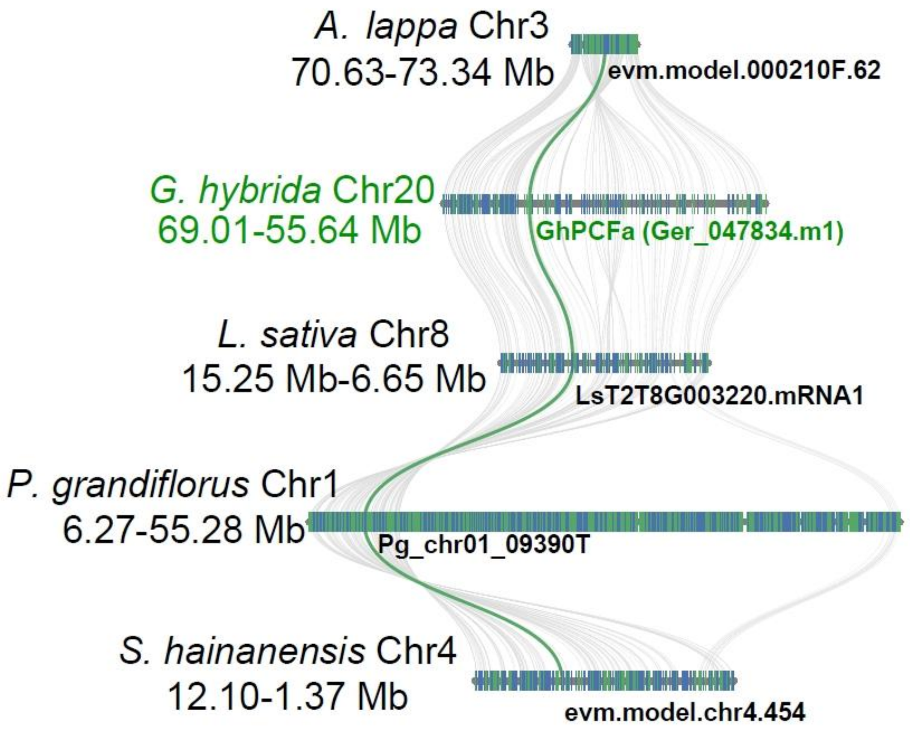

Figure S19 The collinear synteny of *Ger_047834.m1* in PCF gene family among *A. lappa*, *G. hybrida*, *L. sativa*, *P. grandiflorus* and *S. hainanensis*.

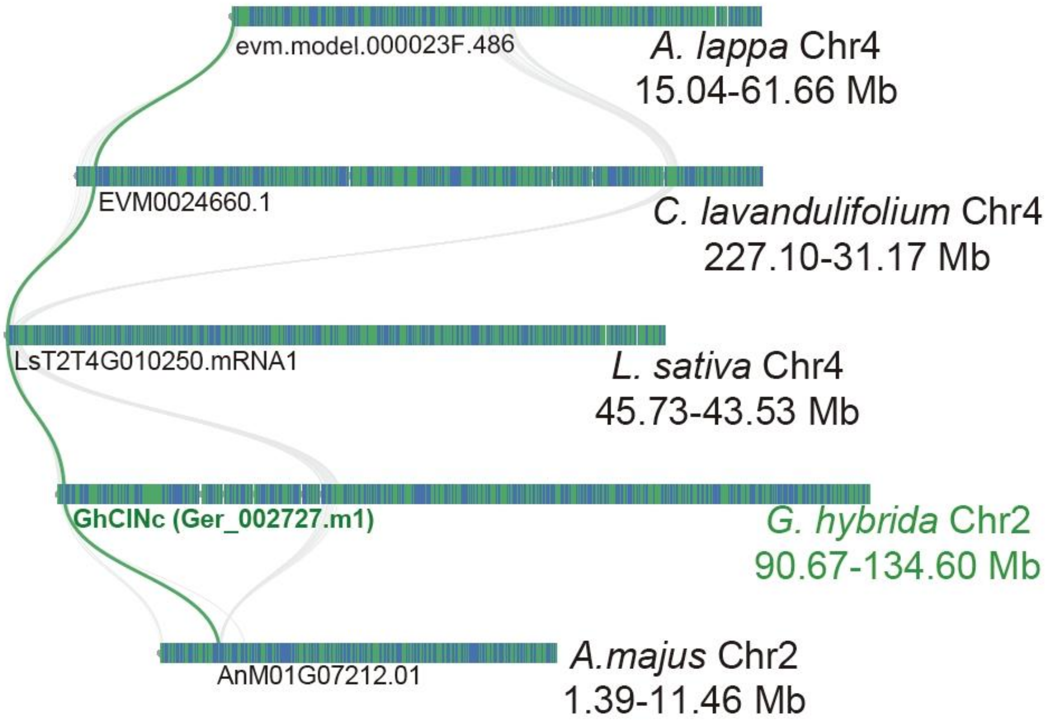

Figure S20 The collinear synteny of *Ger_002727.m1* in CIN gene family among *A. lappa*, *C. lavandulifolium*, *L. sativa*, *G. hybrida* and *A. majus*.

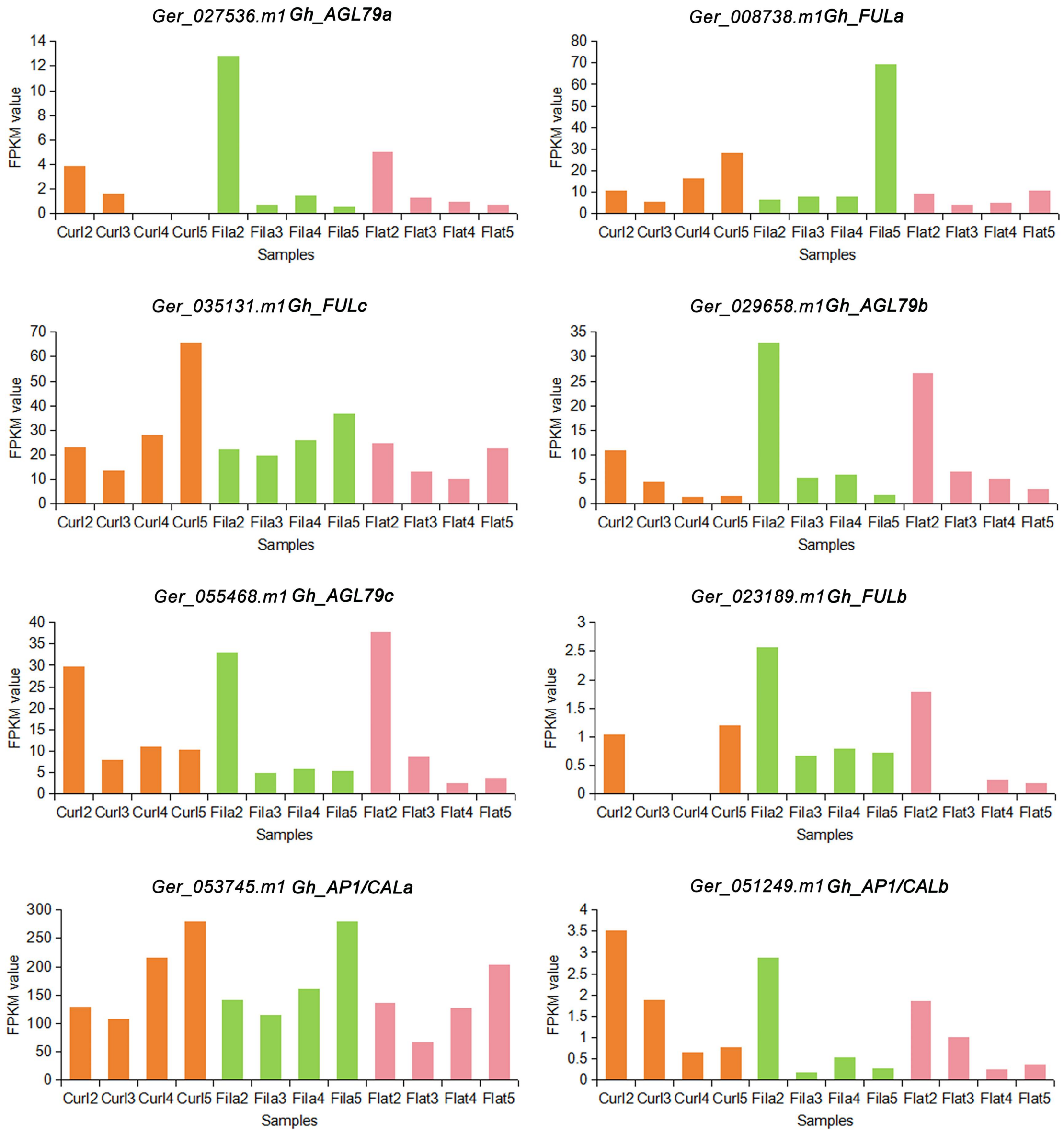

Figure S21 The expression patterns of genes in AP1/FUL subfamily during the development of different types ray florets.

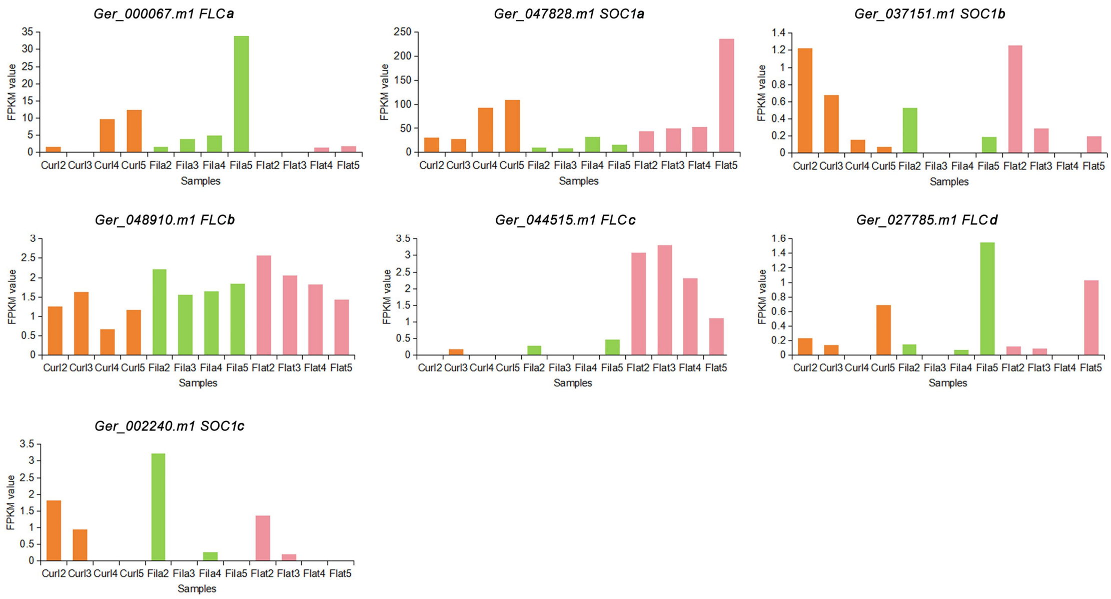

Figure S22 The expression patterns of genes in FLC and SOC1 subfamily during the development of different types ray florets.

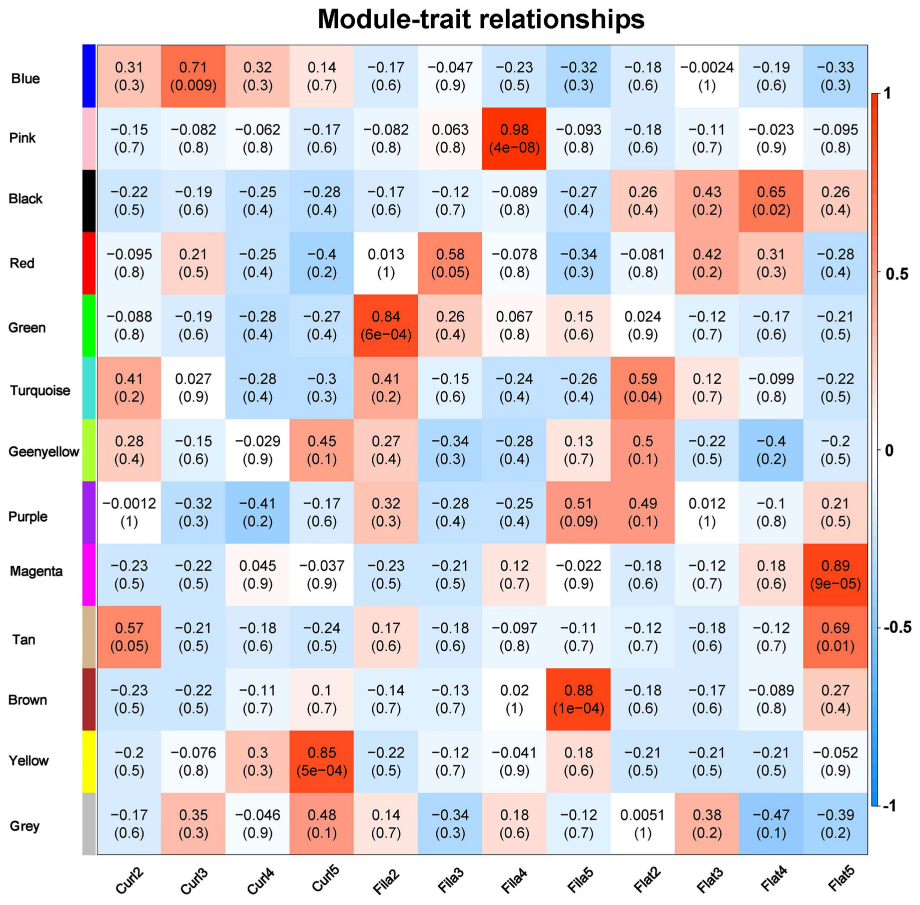

Figure S23 WGCNA for the genes expressed in different ray florets types (curl type, fila type and flat type)

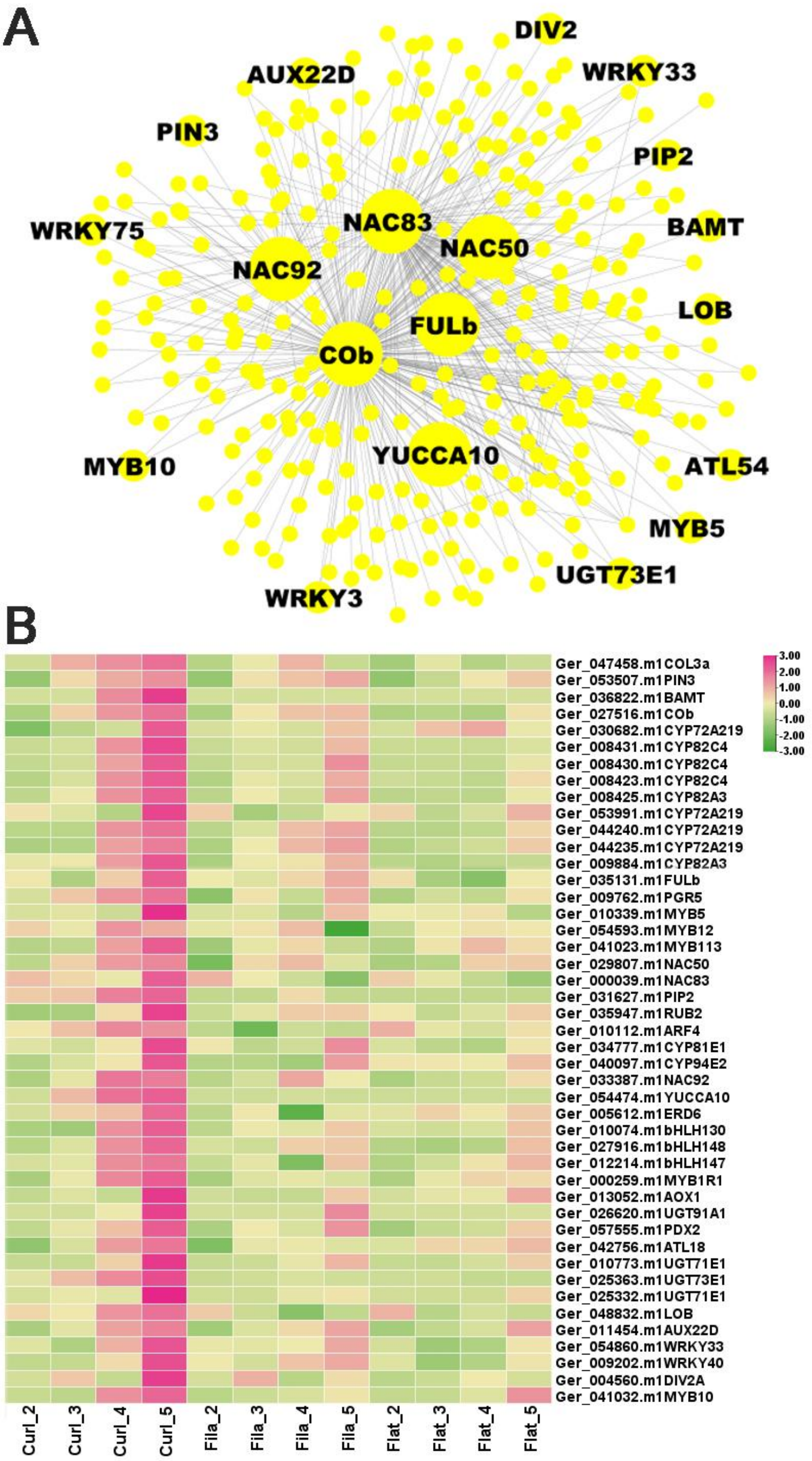

Figure S24 Candidate genes in yellow module which is related to curl ray florets. A, Co-expression network of hub genes enriched in yellow module. B, The heatmap of candidate genes in yellow module. Scale = Log_2FPKM

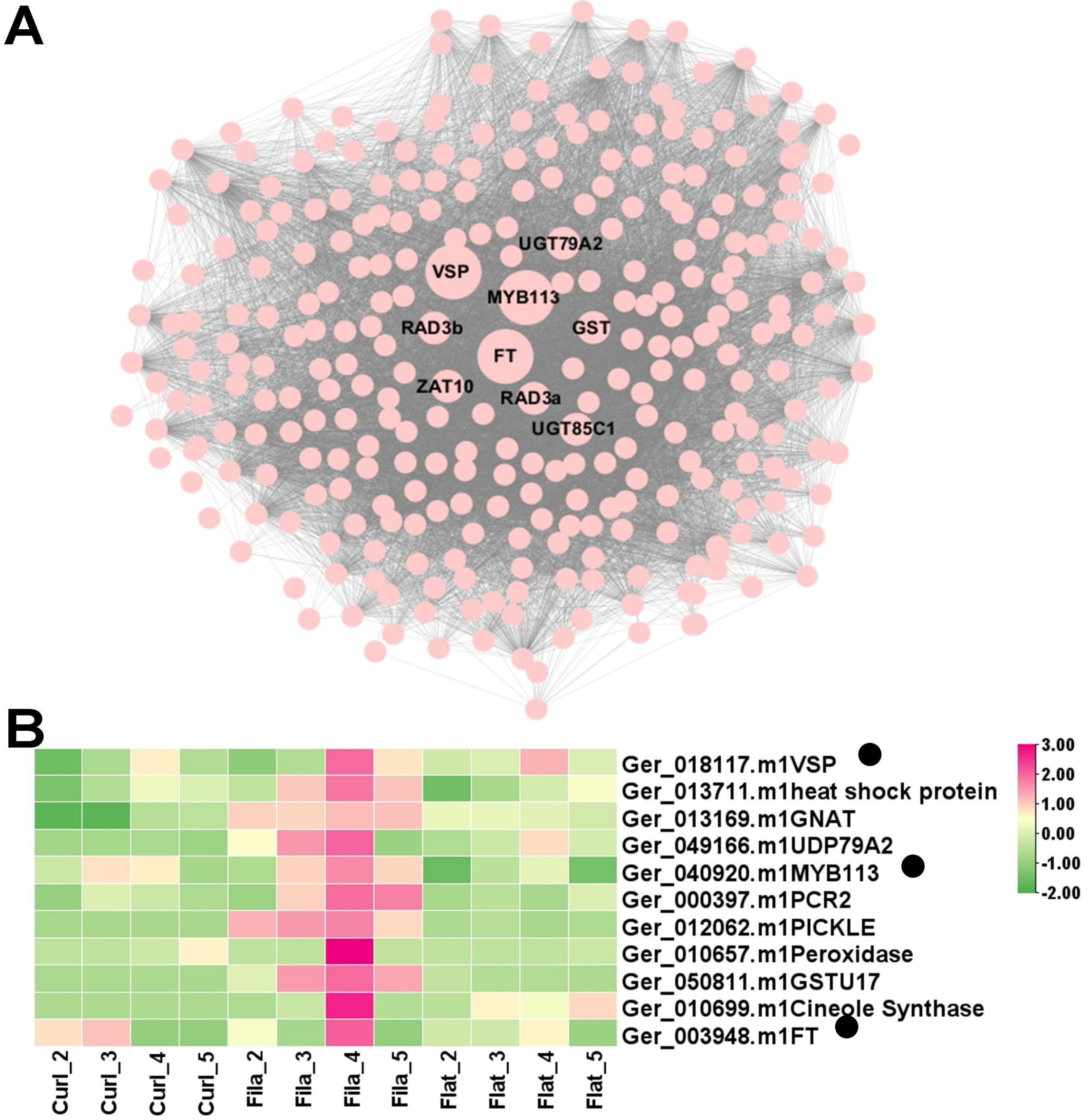

Figure S25 Candidate genes in pink module which is related to fila ray florets. A, Co-expression network of hub genes enriched in pink module. B, The heatmap of candidate genes in pink module. Scale = Log_2FPKM

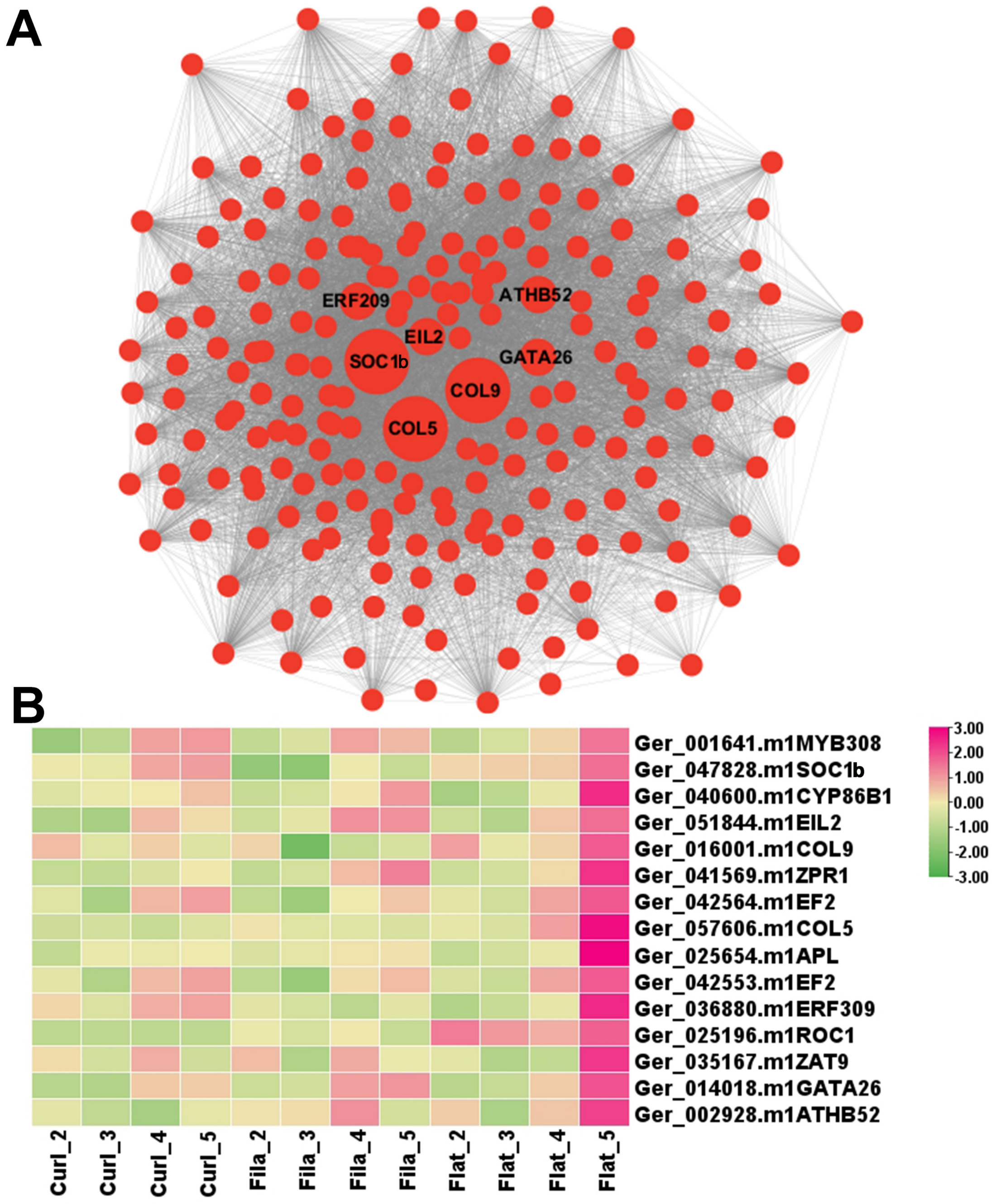

Figure S26 Candidate genes in pink module which is related to flat ray florets. A, Co-expression network of hub genes enriched in red module. B, The heatmap of candidate genes in red module. Scale = Log_2FPKM

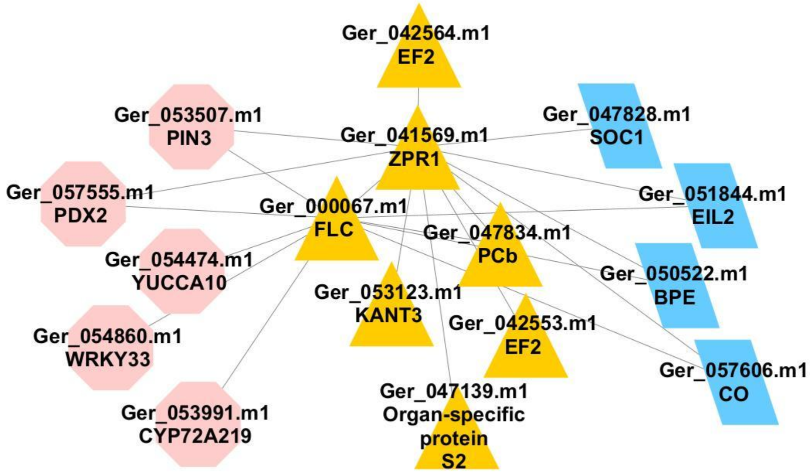

Figure S27 Co-expression network key candidate genes for different types ray florets in *G. hybrida*.

For Review Only

Supplementary Tables

Table S1 Genome assembly of *G. hybrida* 'sh6' and other eight Asteraceae species.

Assembly statistics	Assembly ID	Assembled genome size	Number of chromosomes (n)	Contig N50	Scaffold N50	Complete BUSCO (C)
***G. hybrida* 'hongjixing'**	**Present study**	**2.27**	**25**	**74.18 Mb**	**86.83 Mb**	**96.90%**
A. annua	ASM311234v1	1.79	9	20.14 Kb	104.89 Mb	91.02%
C. cardunculus	CcrdV1	0.73	17	19.39 Kb	125.94 Mb	98.82%
C. lavandulifolium	ASM2254549v1	2.64	9	497 Kb	330.4 Mb	90.64%
C. nankingenese	Chrysanthemum_nankingense_Hbjv0	2.53	9	130.68 Kb	-	80.04%
H. annuus	HanXRQr1.0	3.06	17	414 Kb	178.89 Mb	98.33%
L. sativa	LsT2T	2.59	9	320.7 Mb	-	99.19%
M. micrantha	ASM936387v1	1.79	19	1.1 Gb	131.57 Mb	80.60%
T. kok-saghyz	TaoLin/TK1151	1.04	8	1.28 Mb	100.2 Kb	90.08%

Notes: Genome assembly statistics were generated in the present study. The genome information of other eight Asteraceae species were retrieved from the Asteraceae Multi-omics Information Resource（AMIR）(https://yanglab.hzau.edu.cn/AMIR).

Table S2 The statistics of gap numbers in each chromosomes

Chromosomes	gaps	length (bp)
**Chr2**	0	146,770,359
**Chr3**	0	136,536,331
**Chr5**	0	99,824,314
**Chr8**	0	90,295,468
**Chr10**	0	87,971,955
**Chr15**	0	79,520,236
**Chr21**	0	73,762,387
**Chr22**	0	73,625,018
**Chr1**	1	158,253,583
**Chr6**	1	95,353,495
**Chr7**	1	92,720,290
**Chr12**	1	86,429,945
**Chr14**	1	80,598,569
**Chr16**	1	77,543,417
**Chr17**	1	77,537,785
**Chr18**	1	77,258,371
**Chr19**	1	75,881,110
**Chr20**	1	73,871,957
**Chr23**	1	73,129,117
**Chr11**	2	86,828,582
**Chr13**	2	84,864,606
**Chr25**	2	59,664,893
**Chr9**	3	88,493,545
**Chr24**	3	72,963,010
**Chr4**	10	120,643,462

For Review Only

Table S3 Repeat annotation of *G. hybrida*

Classification			Count	total_repeat	repeat_percent
Retroelements	LINEs	CRE/SLACS	8	347 bp	0.00%
		L2/CR1/Rex	2,231	623,080 bp	0.03%
		RTE/Bov-B	436	45,324 bp	0.00%
		L1/CIN4	4,569	5,463,962 bp	0.23%
	LTR elements	Ty1/Copia	675,754	829,914,660 bp	34.64%
		Gypsy/DIRS1	167,949	211,392,981 bp	8.82%
		Retroviral	11,917	1,653,471 bp	0.07%
DNA transposons		hobo-Activator	10,274	5,655,749 bp	0.24%
		Tc1-IS630-Pogo	599	333,509 bp	0.01%
		Tourist/Harbinger	3,125	1,605,878 bp	0.07%
Rolling-circles			230	151,794 bp	0.01%
Unclassified			1,633,870	590,195,072 bp	24.63%
Total interspersed repeats				2,040,272,531 bp	85.15%
Small RNA			3,807	6,708,289 bp	0.28%
Satellites			1	69 bp	0.00%
Simple repeats			380,129	20,724,084 bp	0.86%
Low complexity			40,434	2,245,865 bp	0.09%

For Review Only

Table S4 The candidate genes that might be involved in ray florets differentiation.

GeneID	Curl_2	Curl_3	Curl_4	Curl_5	Fila_2	Fila_3	Fila_4	Fila_5	Flat_2	Flat_3	Flat_4	Flat_5
Ger_057555.m1PDX2	17.99	22.87	31.14	58.31	15.57	24.11	20.88	41.43	14.74	18.41	19.47	28.96
Ger_054860.m1WRKY33	1.76	0.59	5.17	16.55	2.04	1.86	2.22	5.84	1.42	0.32	0.62	2.49
Ger_054474.m1YUCCA10	0.06	2.29	8.47	11.11	0.00	0.00	0.09	0.00	0.00	0.00	0.00	0.00
Ger_053991.m1CYP72A219	3.17	1.81	0.41	50.81	4.86	0.14	0.80	2.39	4.43	0.63	1.43	7.82
Ger_053507.m1PIN3	12.42	79.59	176.33	280.21	12.25	70.89	118.69	186.19	11.27	29.21	64.19	110.67
Ger_048832.m1LOB	5.51	4.52	11.70	17.24	6.32	2.67	0.91	2.38	8.24	2.04	3.04	2.56
Ger_044728.m1ZFP32	1.45	5.37	64.55	239.71	2.14	3.79	7.14	8.32	4.43	6.59	1.87	2.40
Ger_043229.m1 NAC50	14.11	47.13	53.70	62.09	21.20	25.38	39.89	19.07	10.91	18.31	7.84	16.89
Ger_042756.m1ATL18	1.09	2.86	8.25	11.64	0.82	3.40	3.49	3.09	2.95	4.65	4.62	6.10
Ger_041032.m1MYB10	0.19	0.09	2.46	3.86	0.06	0.24	0.36	0.75	0.15	0.16	0.43	2.38
Ger_041023.m1MYB113	0.63	0.94	17.74	66.68	0.05	3.03	5.89	1.14	0.54	4.42	10.90	5.32
Ger_040097.m1CYP94E2	1.91	5.00	9.22	159.30	2.10	2.15	1.15	40.53	9.57	9.07	7.94	19.81
Ger_036822.m1BAMT	0.00	0.00	9.82	50.75	0.00	0.00	0.00	0.00	0.00	0.00	0.00	0.00
Ger_035947.m1RUB2	234.20	246.63	444.22	1337.52	308.87	370.40	504.00	504.27	415.30	296.46	351.73	497.31
Ger_035131.m1FUL	20.94	11.64	25.96	63.85	20.25	17.77	23.98	34.64	22.80	11.23	8.39	20.51
Ger_035130.m1FUL	20.92	10.74	25.76	61.12	19.74	17.07	21.75	31.21	21.39	9.83	7.86	17.10
Ger_034777.m1CYP81E1	1.22	0.76	2.07	30.68	1.99	0.28	0.74	10.67	0.99	0.28	0.11	2.09
Ger_033387.m1NAC92	0.06	1.63	19.36	17.25	0.30	0.98	8.30	2.05	0.05	0.62	0.84	3.00
Ger_031627.m1PIP2	4.27	5.11	28.08	52.22	0.00	0.00	2.88	0.00	0.00	0.00	0.00	0.00
Ger_027516.m1COL2	1.25	17.29	69.26	152.19	1.25	10.43	23.46	26.98	1.37	1.83	1.29	11.55
Ger_026620.m1UGT91A1	0.00	0.00	0.53	14.47	0.00	0.00	0.00	4.62	0.00	0.00	0.00	0.07
Ger_025363.m1UGT73E1	0.63	3.96	12.02	39.78	0.04	0.20	0.45	0.24	0.15	0.19	0.00	0.02
Ger_025332.m1UGT71E1	0.13	0.49	0.31	23.98	0.02	0.00	0.37	0.76	0.04	0.00	0.00	1.22
Ger_013052.m1AOX1	0.18	0.90	0.24	57.88	0.45	0.25	0.18	3.50	0.51	1.04	1.21	6.88
Ger_011454.m1AUX22D	0.31	2.75	17.37	33.22	0.14	2.36	5.66	16.49	0.20	1.11	2.15	17.95
Ger_011060.m1NIPA3	11.94	4.08	27.13	89.29	5.36	2.20	3.71	39.69	0.50	0.00	1.09	0.88
Ger_010773.m1UGT71E1	0.00	0.05	1.69	23.80	0.03	0.10	0.67	2.96	0.02	0.00	0.27	1.83
Ger_010339.m1MYB5	1.37	1.76	1.00	43.85	1.41	1.64	0.54	5.06	2.25	1.98	2.74	0.50
Ger_010074.m1bHLH130	4.82	4.16	25.83	42.76	5.87	9.91	8.85	14.31	7.80	7.15	8.03	15.29
Ger_009762.m1PGR5	9.51	34.43	95.28	190.84	1.34	19.75	6.64	56.87	3.53	8.34	6.01	20.45
Ger_009202.m1WRKY40	0.90	1.02	3.53	32.60	2.74	1.40	5.42	8.13	1.83	0.11	0.54	3.05
Ger_008970.m1CYP71A8	4.45	7.53	78.30	188.53	0.84	1.53	0.30	1.60	2.15	1.42	5.22	28.88
Ger_008967.m1CYP71A8	4.34	9.01	137.51	280.14	0.10	0.00	0.00	2.85	1.48	1.84	7.24	40.08
Ger_008965.m1CYP71A21	4.45	5.58	50.32	127.54	0.83	0.22	0.47	2.26	2.50	1.33	3.37	19.65
Ger_005612.m1ERD6	26.89	40.31	46.52	91.64	22.22	34.40	9.85	29.52	29.39	41.11	34.29	42.70
Ger_004560.m1DIV2A	0.65	1.83	0.47	8.22	0.70	2.45	0.33	1.39	0.36	0.54	1.14	0.69
Ger_002727 CIN	29.97	89.52	68.14	61.96	26.89	17.38	11.16	7.69	23.80	34.53	1.69	4.35
Ger_000039.m1NAC83	17.57	12.57	8.79	64.89	20.67	9.40	5.74	1.80	14.28	8.25	4.99	2.68
Ger_018117.m1VSP	1.26	4.98	25.77	5.70	2.77	5.67	116.16	29.00	9.69	11.56	51.22	11.51
Ger_012988.m1GAS	0.00	0.00	0.00	0.06	0.16	283.94	0.38	1997.27	0.00	0.35	242.82	811.86
Ger_041325.m1ATL8	17.56	8.39	7.72	34.05	17.17	5.44	23.42	143.05	10.07	12.74	10.28	39.68
Ger_028926.m1UGT92A1	29.43	50.28	37.55	35.35	28.26	54.54	57.06	114.61	30.01	47.65	56.22	45.66
Ger_028378.m1CYPb7	0.00	0.05	0.14	0.83	5.94	6.30	16.22	93.93	0.84	0.00	0.57	8.03
Ger_057698.m1SWEET7	0.17	0.24	0.12	2.47	0.00	2.91	14.12	75.98	0.00	0.00	0.50	15.90
Ger_008738.m1AGL8	8.44	3.26	14.19	25.73	4.13	5.60	5.60	67.15	7.05	1.89	2.82	8.43
Ger_047139.m1Organ-specific protein S2	0.00	0.00	0.00	0.00	7.60	1.12	0.04	56.39	0.00	0.00	0.04	19.37
Ger_053123.m1KANT3	39.69	13.70	7.94	12.73	22.57	8.64	13.92	53.57	23.02	11.17	8.19	26.11
Ger_003782.m1ATL32	4.52	5.89	4.69	11.72	6.05	5.58	9.14	45.89	6.57	8.03	7.80	11.11
Ger_033181.m1Expansin-A9	0.00	0.00	0.00	0.00	0.06	0.00	0.98	27.95	0.00	0.00	0.00	0.00
Ger_035469.m1bHLH116	47.34	15.11	13.78	6.50	24.12	8.88	12.44	83.57	28.10	14.38	6.89	8.80
Ger_000067.m1FLCa	0.48	0.00	8.67	11.37	0.49	2.91	3.87	32.93	0.00	0.00	0.30	0.83
Ger_047834.m1PCb	23.69	11.30	24.69	14.70	24.09	68.52	30.41	6.32	27.36	8.13	19.66	7.07
Ger_003948.m1FT	5.22	7.33	0.00	0.00	3.32	0.33	21.80	0.00	1.35	1.28	4.16	0.11
Ger_008738.m1FULc	8.44	3.26	14.19	25.73	4.13	5.60	5.60	67.15	7.05	1.89	2.82	8.43
Ger_048774.m1GEGb	4.47	20.67	0.00	0.01	15.73	0.48	47.38	0.00	0.00	39.23	27.18	14.66
Ger_057723.m1bHLH014	2.07	0.41	0.00	0.02	1.23	0.00	0.00	0.00	28.01	0.11	0.00	0.02
Ger_057606.m1CO	0.12	0.21	0.02	0.35	0.95	0.44	0.46	0.32	0.59	0.31	2.72	15.13
Ger_051844.m1EIL2	25.75	24.52	58.06	48.19	33.04	40.11	74.17	74.32	32.60	27.20	54.70	91.19
Ger_050522.m1BPE	15.05	19.44	87.88	28.70	12.55	25.14	54.12	23.09	13.21	28.44	104.65	152.12
Ger_047828.m1SOC1	23.89	21.05	84.86	101.14	1.94	1.51	24.48	8.43	36.96	42.93	45.07	228.37
Ger_042564.m1EF2	4.13	1.96	7.86	9.38	2.72	1.60	5.13	6.86	4.36	3.38	9.17	16.50
Ger_042553.m1EF2	3.57	1.89	5.96	7.21	2.28	1.33	4.65	5.94	3.14	2.61	6.98	11.98
Ger_041569.m1ZPR1	0.57	0.49	1.22	2.47	0.47	1.09	4.84	11.52	0.79	1.03	3.07	31.66
Ger_040600.m1CYP86B1	6.88	9.40	10.64	18.70	4.19	5.83	10.89	32.75	1.83	3.20	8.59	147.33
Ger_036880.m1ERF309	2.13	1.17	3.21	3.60	1.30	1.01	0.53	1.31	0.40	0.74	1.46	10.52
Ger_035167.m1ZAT9	2.81	1.77	3.96	1.67	3.53	1.09	4.02	2.36	2.15	1.12	1.41	8.71
Ger_034015.m1MYB9A	0.08	0.11	0.04	0.13	0.00	0.04	0.09	0.00	1.13	0.00	0.03	0.19
Ger_033604.m1SEUe	5.33	3.52	3.62	3.95	2.27	0.68	0.96	1.47	28.42	21.58	15.22	12.61
Ger_029769.m1MYB44	0.40	0.13	0.00	0.00	1.04	0.03	0.28	0.00	10.14	0.41	0.07	0.00
Ger_028585.m1WRKY15	0.23	0.30	0.00	0.08	2.05	0.15	0.03	0.49	0.17	0.23	0.03	3.48
Ger_025654.m1APL	0.04	0.71	0.68	0.82	0.37	0.58	0.94	0.98	0.06	0.28	0.66	12.67
Ger_025196.m1ROC1	0.00	0.00	0.00	0.00	1.13	0.47	1.34	0.12	7.26	4.82	3.54	10.27
Ger_020782.m1FAF3	2.82	1.38	0.03	1.24	1.51	0.34	0.64	1.64	0.71	9.39	0.77	0.42
Ger_016001.m1CO9	22.54	12.17	19.15	11.74	18.30	2.60	8.54	10.94	28.74	13.32	18.63	53.31
Ger_014018.m1GATA26	0.75	0.65	2.47	2.42	1.06	1.20	3.49	3.84	0.76	0.96	2.42	6.33
Ger_009151.m1LEAFY	0.32	0.05	0.04	0.10	0.60	0.39	0.13	0.00	5.01	0.05	0.02	0.00
Ger_008200.m1LOB38	0.00	0.55	0.22	0.42	0.59	0.89	0.82	0.72	0.88	0.95	2.57	0.79
Ger_004900.m1PCFa	28.73	16.65	26.18	28.71	33.38	34.52	31.62	17.00	63.66	25.83	20.24	22.72
Ger_003986.m1ERF1B	0.00	0.00	0.00	0.00	0.09	0.00	0.00	0.00	1.22	0.00	0.00	0.00
Ger_003107.m1MYB44	0.00	0.27	0.00	0.00	0.20	0.32	0.62	0.10	0.00	0.51	1.40	0.09
Ger_002928.m1ATHB52	0.68	0.25	0.00	0.72	0.98	1.07	2.27	0.48	1.26	0.04	1.34	4.30
Ger_001641.m1MYB308	14.92	26.50	146.90	161.36	29.44	42.54	145.28	111.40	24.53	39.79	81.39	247.90
Ger_000335.m1MYB106	0.33	0.00	0.00	0.00	0.32	0.00	0.00	0.00	2.85	0.00	0.00	0.00
